# Functional Morphology of the Thorax of the Click Beetle *Campsosternus auratus* (Coleoptera, Elateridae), with an Emphasis on Its Jumping Mechanism

**DOI:** 10.3390/insects13030248

**Published:** 2022-02-28

**Authors:** Yongying Ruan, Mengna Zhang, Robin Kundrata, Lu Qiu, Siqin Ge, Xingke Yang, Xiaoqin Chen, Shihong Jiang

**Affiliations:** 1 Plant Protection Research Center, Shenzhen Polytechnic, Shenzhen 518055, China; yongyingruan@szpt.edu.cn (Y.R.); mengnazhang@szpt.edu.cn (M.Z.); sjiang@szpt.edu.cn (S.J.); 2 Key Laboratory of Zoological Systematics and Evolution, Institute of Zoology, Chinese Academy of Sciences, Beijing 100101, China; yangxk@ioz.ac.cn; 3 Department of Zoology, Faculty of Science, Palacky University, 17. Listopadu 50, 771 46 Olomouc, Czech Republic; robin.kundrata@upol.cz; 4 Engineering Research Center for Forest and Grassland Disaster Prevention and Reduction, Mianyang Normal University, Mianxing West Road, Mianyang 621000, China; 123church@163.com; 5 Guangdong Public Laboratory of Wild Animal Conservation and Utilization, Institute of Zoology, Guangdong Academy of Sciences, Guangzhou 510260, China

**Keywords:** click beetles, 3D reconstruction, musculature, function, clicking, locking, clamping

## Abstract

**Simple Summary:**

Click beetles are well-known for the specialized thoracic structure, which they can click to thrust themselves into the air and to right themselves. Several aspects of their jumping mechanism were still not entirely clear prior to this study. We utilized traditional dissection, 3D virtual dissection, and high-speed filming techniques to investigate the functional morphology of their thorax. Our results show several new insights into their extraordinary clicking and jumping mechanisms.

**Abstract:**

We investigated and described the thoracic structures, jumping mechanism, and promesothoracic interlocking mechanism of the click beetle *Campsosternus auratus* (Drury) (Elateridae: Dendrometrinae). Two experiments were conducted to reveal the critical muscles and sclerites involved in the jumping mechanism. They showed that M2 and M4 are essential clicking-related muscles. The prosternal process, the prosternal rest of the mesoventrite, the mesoventral cavity, the base of the elytra, and the posterodorsal evagination of the pronotum are critical clicking-related sclerites. The destruction of any of these muscles and sclerites resulted in the loss of normal clicking and jumping ability. The mesonotum was identified as a highly specialized saddle-shaped biological spring that can store elastic energy and release it abruptly. During the jumping process of *C. auratus*, M2 contracts to establish and latch the clicking system, and M4 contracts to generate energy. The specialized thoracic biological springs (e.g., the prosternum and mesonotum) and elastic cuticles store and abruptly release the colossal energy, which explosively raises the beetle body in a few milliseconds. The specialized trigger muscle for the release of the clicking was not found; our study supports the theory that the triggering of the clicking is due to the building-up of tension (i.e., elastic energy) in the system.

## 1. Introduction

Various groups of insects can jump in an explosive manner. In recent decades, the understanding of their jumping mechanisms and related biological structures has advanced significantly, such as in the case of fleas [1,2], locusts [3,4,5], froghoppers [6], lantern bugs [7], and beetles [8,9,10].

Click beetles (Elateridae) are well known for their energetic clicks, which can thrust them into the air and allow them to right themselves. In contrast to most other jumping insects, which utilize the hind legs, they jump using their unique thoracic structure, which probably appeared more than 200 million years ago [11,12]. Their jumps are caused by a very rapid jack-knifing of the anterior and posterior part of the body, which raises a beetle’s center of gravity in a few milliseconds [13].

Preliminary investigations on the jump of Elateridae have been made by Binaghi [14] and d’Aguilar [15]. The thoracic muscles of *Elater sanguineus* L. and *Selatosomus aeneus* (L.) were investigated by Larsén [16]. The jumping-related structures and jumping mechanism of *Athous haemorrhoidalis* (F.) were studied in detail by Evans [13]. Subsequently, Evans [17] studied the jumping mechanics and energetics of the same species. Several recent studies further significantly advanced the understanding of the mechanics and dynamics of the click-beetle jumps. Ribak and Weihs [18], in addition to Ribak et al. [19], revealed that the jumping technique of Elateridae is constrained by their body shape. They showed that click beetles cannot control their body orientation during the landing phase and cannot perform long-distance jumps. Ribak et al. [20] analyzed the effect of natural substrates on click beetles’ jumping height. Bolmin et al. [21,22] studied and described the dynamics and mechanics of each phase of the jump of Elateridae and summarized the jumping mechanism. They also studied and modeled the fast unbending and oscillation in the jumps of *Elater abruptus* (Say) [23].

Several studies explored bionic designs mimicking the morphology of Elateridae. For instance, Evans [13] designed a two-dimensional hopping model of a clicking beetle based on the shape of the sagittal section of *Athous haemorrhoidalis*; Fukushima and Kawaguchi [24], in addition to Chen et al. [25], developed jumping robots inspired by the jumping style of Elateridae (but the morphology of the thoracic structures of Elateridae was not mimicked).

Despite the studies mentioned above, there are still many aspects of the jumps of Elateridae that are not clear: for instance, (1) the functions and detailed morphology of the thoracic muscles and sclerites involved in the clicking; (2) the trigger of the clicking; (3) how the brain and nerve system sustain the impact caused by the clicking; and (4) if all groups of click beetles, as well as other clicking elateroids, share precisely the same clicking mechanism.

*Campsosternus auratus* (Drury) is one of the most common and widely distributed click beetles in the Oriental region [26]. Its thorax is characterized by an elongated prosternal process, deep mesoventral cavity, and well-developed pronotal posterior angles. Using the unique thoracic structures and jumping mechanism typical for click beetles it leaps into the air and right its body by chance. In this work we studied the thoracic structures of *C. auratus* using micro-CT and 3D reconstruction techniques, as well as the functions of the thoracic muscles and sclerites in the clicking and thoracic interlocking behaviors. Furthermore, the jumping process and features of *C. auratus* were investigated with high-speed filming technique.

## 2. Materials and Methods 

**Figure 1:**
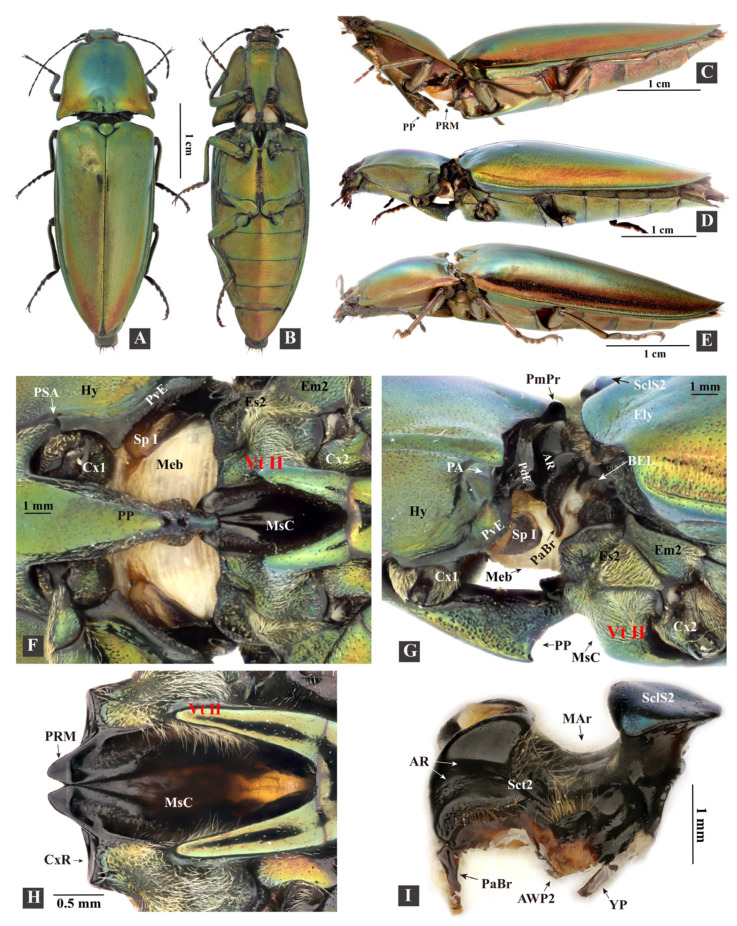
Habitus and exoskeleton of *Campsosternus auratus*. See Section 2 ‘Materials and Methods’ for abbreviations. (**A**) dorsal view, resting position. (**B**) ventral view, latching position; the prosternal process (PP) is latched onto the prosternal rest of the mesoventrite (PRM). (**C**) lateral view of a live individual in a back-arched position preparing to latch onto the PP and PRM. (**D**) lateral view of a specimen in latching position, PP is latched onto the PRM. (**E**) lateral view of a live individual in resting position. (**F**) ventral view of promesothoracic gap, zoomed-in from inset (**B**). (**G**) lateral view of promesothoracic gap, zoomed-in from inset (**D**); **Meb** indicates the promesothoracic intersegmental membrane. (**H**) ventral view of the mesoventrite. (**I**) lateral view of the mesonotum.

**Figure 2:**
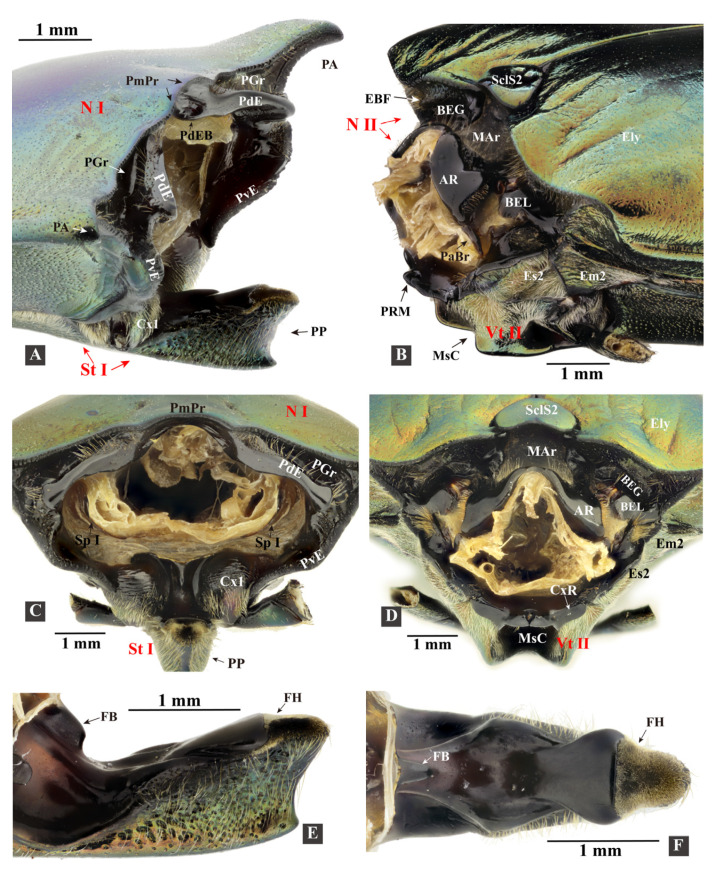
Exoskeleton of *Campsosternus auratus*. See Section 2 ‘Materials and Methods’ for abbreviations. (**A**) posterolateral view of the posterior part of the prothorax. (**B**) anterolateral view of the mesothorax. (**C**) caudal view of the prothorax. (**D**) frontal view of the mesothorax. (**E**) lateral view of the prosternal process. (**F**) dorsal view of the prosternal process.

The specimen information for *Campsosternus auratus* and other Elateriformia species is listed in Table 1 and Supplementary File S1. The Elateridae classification follows that laid out by Kundrata et al. [27] and Douglas et al. [28]. The voucher specimens are preserved in the Plant Protection Research Center, Shenzhen Polytechnic, Shenzhen, Guangdong, China. The digital images in Figure 1 and Figure 2 were taken with a Canon D800 camera attached to a Canon MP-E 65 mm lens. Figure plates were prepared with Photoshop CS5 (Adobe, San Jose, CA, USA) and Illustrator CS5 (Adobe, San Jose, CA, USA).

### 2.1. Terminology

Morphological terms are largely based on those of Larsén [16]; however, several other studies were examined for either currently used terms or for alternative terminology (see below). Abbreviations and terms used in Figure 1, Figure 2, Figure 3, Figure 4, Figure 5, Figure 6, Figure 7, Figure 8, Figure 9, Figure 10, Figure 11, Figure 12 and Figure 13 are listed and explained below. 

The ‘**clicking mechanism**’ (clicking) and ‘**jumping mechanism**’ (jumping) are slightly different in process. (1) The ‘clicking mechanism’ can be performed whenever the click beetles’ prothorax is free to move, even when natural enemies or humans catch them; it includes **latching**, **loading**, and **releasing** phases. (2) The ‘jumping mechanism’ only happens when the click beetles lie on the ground, inverted, and click to thrust them into the air, including the **latching**, **loading**, **take-off**, and **airborne** phases. The separation of these phases is adapted from Bolmin et al. [21,23]. (3) The ‘**triggering**’ of the clicking happens between the loading and take-off phases, when the accumulation of tension and the deformation of thoracic structures make the latching system yield. 

The ‘**thoracic hinge**’ consists of the posterodorsal evagination of the pronotum (PdE) and the anterolateral region of the mesonotum (AR, Figure 1I and Figure 13D). The thoracic hinge center is defined as the thoracic ‘**pivot**’, which is situated in the hollow center of the AR.

The ‘**back arching**’ (sensu [13]) refers to the bending movement dorsad of the click beetle’s body in the latching phase. The ‘**jack-knifing**’ (sensu [13]) refers to the click beetle’s abrupt bending movement ventrad in the release or take-off phase.

The ‘**fulcrum**’ is a physics term that refers to the supporting point of a lever. In the loading phase of the clicking mechanism, when the prosternal process is pushed ventrally, the pronotosternal articulation (PSA) acts as a fulcrum.

**1AT……8AT** = abdominal tergites 1–8; **1Pm/2Pm/3Pm** = first/second/third phragma of the thorax; **3ASt……7ASt** = abdominal sternites 3–7, equivalent to abdominal ventrites 1–5; **3Pm-ML**/**3Pm-LP** = median lobe/lateral process of the third phragma; **Abd** = abdomen; **Act** = acetabulum; **Alc** = alacrista of the metanotum [29]; **AmE** = anteromedium emargination of the mesonotum; **APm** = abdominal ventral phragma [30,31]; **AR** = anterolateral region of the mesonotum, with a highly smooth surface; **PaBr** = prealar bridge of the mesonotum, also known as the prealar arm in [29]; **AVA** = anteroventral angle of the mesoventral cavity [32]; **AWP2/AWP3** = anterior notal wing process of the mesonotum/metanotum; **Ax1**/**Ax2**/**Ax3** = (first, second, and third) axillary sclerite; **AxC** = axillary cord; **Ba** = basalar sclerite; **BaD** = basalar disc; **BEG** = basal elytral groove [13]; **BEL** = basal lobe of the elytron [16], also known as the ‘elytral root’ in [33,34]; **BSc** = subcostal basivenale [35]; **Cd** = condyle; **Cl** = anterior collar of the pronotum (i.e., inflected anterior margin of the pronotum); **Crpl** = cryptopleuron, equivalent to the endopleuron; **Cu** = elastic cuticle; **Cv1/Cv2** = cervical sclerite 1/2; **Cx1**/**Cx2**/**Cx3** = pro-/meso-/metacoxa; **CxP** = metacoxal plate; **CxR** = procoxal rest of the mesoventrite [36], also known as the ‘anterior articulating surface’ in [37]; **Dc** = metathoracic discrimen [36]; **EB** = elytra base [38]; **EBF** = flange of the elytral base [13]; **EBP** = mesal process of the elytral base, following Friedrich and Beutel [38]; **Ely** = elytron; **Em2/Em3** = mesepimeron/metepimeron; **Epi** = elytral epipleuron; **Es2/Es3** = mesanepisternum/metanepisternum; **F1** = prothoracic furca; **F2** = mesothoracic furca, equivalent to the mesendosternite; **F3** = metathoracic furca, equivalent to the metendosternite; **FB** = profurcal base or prosternal furcal base, also known as the ‘bumper’ in [13]; **Fe** = femur; **FH** = friction hold [13], a lowered area on the posterodorsal end of the prosternal process, also known as the ‘peghold’ in [13]; **FP** = furcal pit; **H** = head; **HP** = humeral plate [35], also known as the ‘costa’ in [16] and the ‘basicostale’ in [29]; **Hy** = hypomeron; **I** = insertion of muscle; **IAM** = inflected anterior margin of the mesonotum; **LA** = lateral arm of the furca [39]; **LC** = lateral carina [40]; **LC-i** = internal trace of the lateral carina; **LGr** = lateral groove of the mesonotum; **PRM** = prosternal rest of the mesoventrite [36], i.e., the anteromedian extension of the mesoventrite, also known as the ‘mesosternal lip’ and ‘lip of the mesosternum’ in [13]; **M1/M2……M85** = muscles 1–85 [29]; **MAr** = median-arched area of the mesonotum; **Meb** = membrane; **MGr** = median groove of the metanotum; **MRMn** = median ridge of the mesonotum, also known as the ‘median phragma’ in [38]; **MRMs** = median ridge of the metaventrite; **MsC** = mesoventral cavity [36], also known as the ‘mesosternal cavity‘ [40] and the ‘mesosternal fossa’ [32]; **MsP** = mesoventral processes [36]; **MtP** = median metasternal processes; **N I/N II/N III** = pro-/meso-/metanotum; **O** = origin of muscle; **PA** = posterior angle of the pronotum; **PCP** = pleural coxal process; **PdE/PvE** = posterodorsal/posteroventral evagination of the pronotum; **PdEB** = the anteromesal bulge part of the **PdE**, sensu the ‘knob‘ on the underside of the posterior margin of the pronotum in [13]; **PE** = posterior evaginations of the pronotum [16], also known as the ‘pronotal flange’ in [13], consisting of the **PdE** and **PvE**; **PGr** = posterodorsal groove of the pronotum, situated above the posterodorsal evagination; **PlA** = pleural arm of the meso-/metapleuron; **PlR** = pleural ridge of the meso-/metapleuron; **PlS** = pleuro suture; **PlWP2/PlWP3** = pleural wing process of the mesothorax/metathorax; **PMG** = promesothoracic gap (the gap between the pro- and mesothorax); **PmPr** = posteromedial part of the pronotum; **Pn3** = postnotum of the metathorax; **PP** = prosternal process; **Pra** = prealar sclerite of the metathorax, consisting of an externally visible isolated sclerite and the internal mushroom-shaped plate; **Prs3** = metathoracic prescutum; **PSA** = pronotosternal articulation; **PScl2** = posterior scutellum of mesonotum; **PsS** = pronotosternal suture; **PWP2/PWP3** = posterior notal wing process of the mesonotum/metanotum; **Sa** = subalar sclerite; **Scl2/Scl3** = meso-/metascutellum; **SclS2** = mesoscutellar shield; **Sct2/Sct3** = mesoscutum/metascutum; **Sp I/Sp II** = prothoracic/mesothoracic spiracle; **SpA1**–**SpA8** = abdominal spiracle 1–8; **SpR** = sternopleural ridge; **SpS** = sternopleural suture; **SSR3** = scutoscutellar ridge of the metanotum; **St I** = prosternum; **Stk** = stalk of the metathoracic furca [39], equivalent to the basal part of the metaendosternite; **Tn** = trochantin; **Tr** = trochanter; **VF** = ventral flange of the metafurca; **Vt II/Vt III** = mesoventrite/metaventrite [36], known as the mesosternum/metasternum in earlier works; and **YP** = yoke plate [29].

### 2.2. Dissection Methods

Both ethanol-preserved and dry specimens were dissected under a Nikon SMZ645 stereomicroscope, using an ophthalmic surgical microscissor and forceps; three specimens used for the examination of the skeleton were immersed in 10% NaOH solution for 24 h before dissection. Specimens were cut in half along the sagittal plane to expose the internal muscles. Subsequently, the muscles were removed one-by-one, to reveal the hidden structures.

### 2.3. Micro-CT Scanning and 3D Reconstructions 

**Figure 3:**
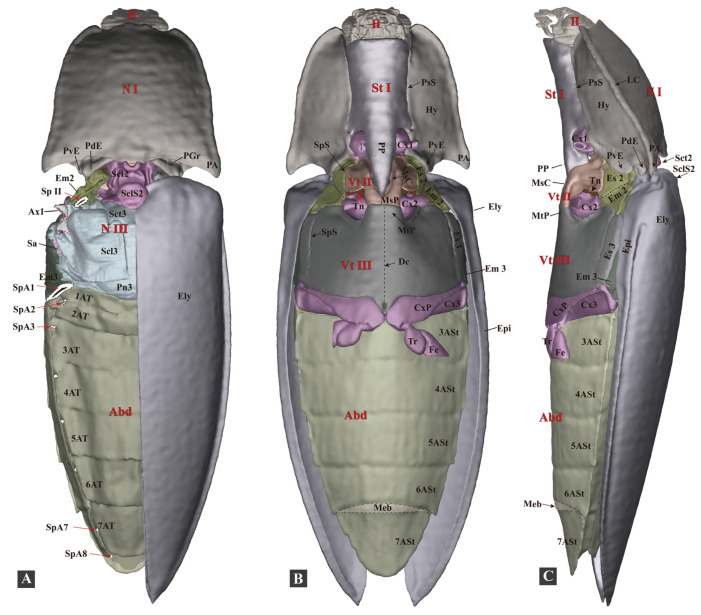
Three-dimensional reconstructions of the exoskeleton of *Campsosternus auratus*. The specimen is in a resting position; for technical information and abbreviations, see Section 2 ‘Materials and Methods’. (**A**) Dorsal view of habitus; left elytron is removed to show the structures beneath; and the Ax1 of the mesothorax and the spiracles were edited in Photoshop, shown in dotted lines. (**B**) Ventral view of habitus. (**C**) Lateral view of habitus.

**Figure 4:**
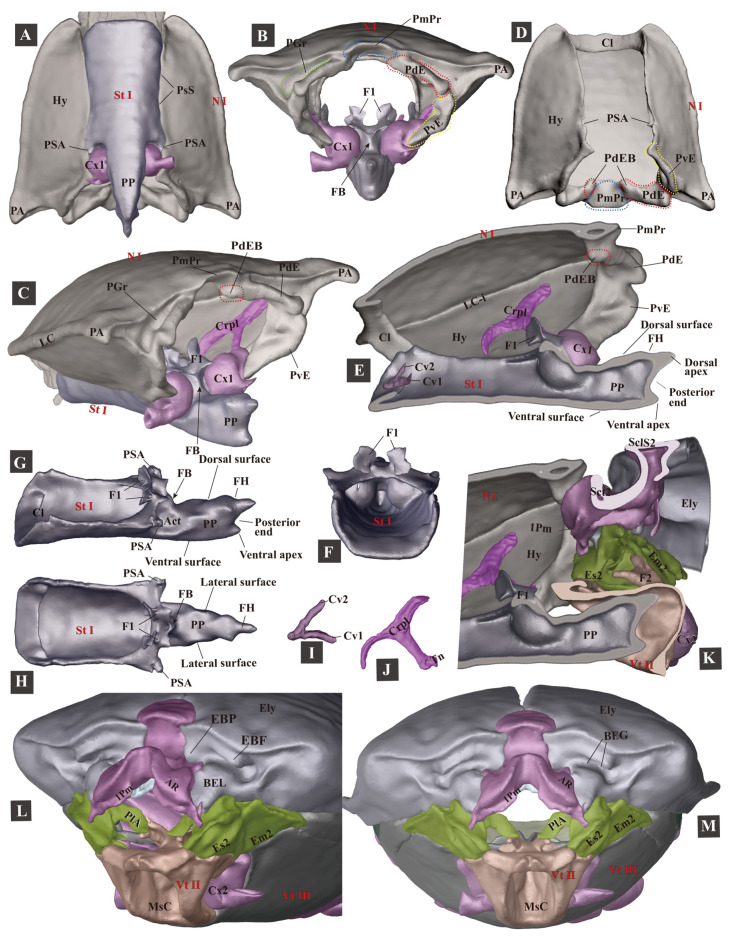
Three-dimensional reconstructions of the pro- and mesothoracic skeleton of *Campsosternus auratus*. For abbreviations, see Section 2 ‘Materials and Methods’. (**A**–**C**) ventral, caudal, and posterolateral views of the prothorax. (**D**) ventral view of the pronotum. (**E**) mesal view of the prothorax cut in sagittal plane. (**F**–**H**) frontal, lateral, and dorsal views of the prosternum. (**I**) the right cervical sclerites, dorsal view, with the anterior part facing left. (**J**) cryptopleuron and trochantin, mesal view. (**K**) mesal view of mesothorax cut in the sagittal plane. (**L**,**M**) anterolateral and frontal views of the mesothorax.

**Figure 5:**
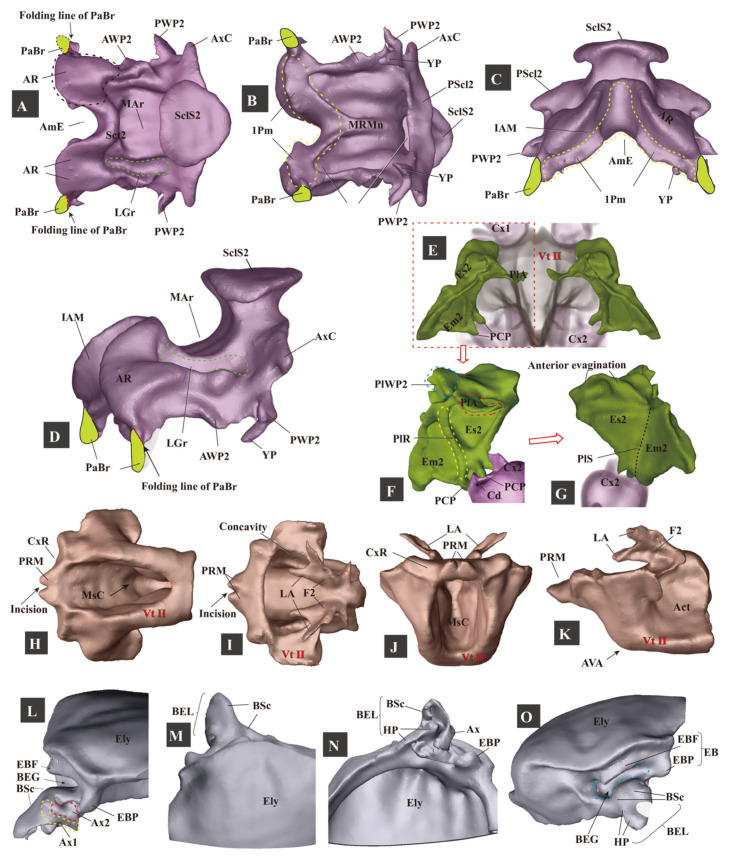
Three-dimensional reconstructions of the mesothoracic skeleton of *Campsosternus auratus*. For technical abbreviations, see Section 2 ‘Materials and Methods’. (**A**–**D**) dorsal, ventral, frontal, and anterolateral views of the mesonotum; PaBr is edited and shown in green color; 1Pm is marked with a yellow dashed line. (**E**) dorsal view of the mesopleura. (**F**) internal view (mesal view) of the left mesopleuron. (**G**) external view (lateral view) of the left mesopleuron. (**H**–**K**) ventral, dorsal, frontal, and lateral views of the mesoventrite. (**L**–**O**) mesal, dorsal, ventral, and frontal views of the basal part of the right elytron.

**Figure 6:**
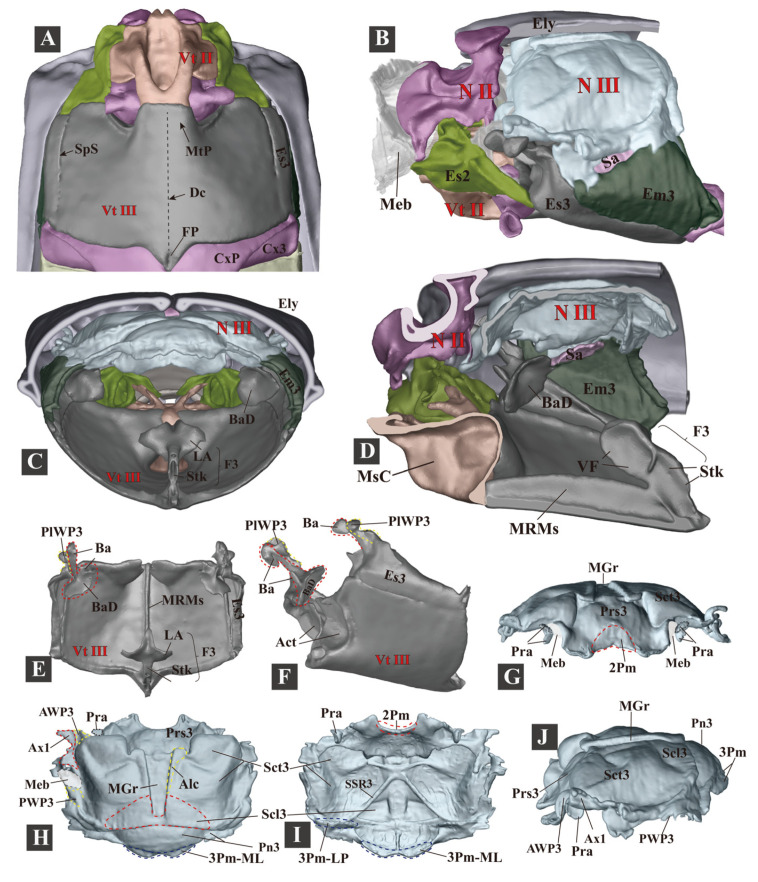
Three-dimensional reconstructions of the metathoracic skeleton of *Campsosternus auratus*. For abbreviations, see Section 2 ‘Materials and Methods’. (**A**) ventral view of the meso- and metathorax. (**B**) lateral view of the meso- and metathorax; the left elytron is removed. (**C**) caudal view of the meso- and metathorax. (**D**) mesal view of the meso- and metathorax cut in the sagittal plane. (**E**,**F**) dorsal and lateral views of the metaventrite. (**G**–**J**) frontal, dorsal, ventral, and lateral views of the metanotum.

**Figure 7:**
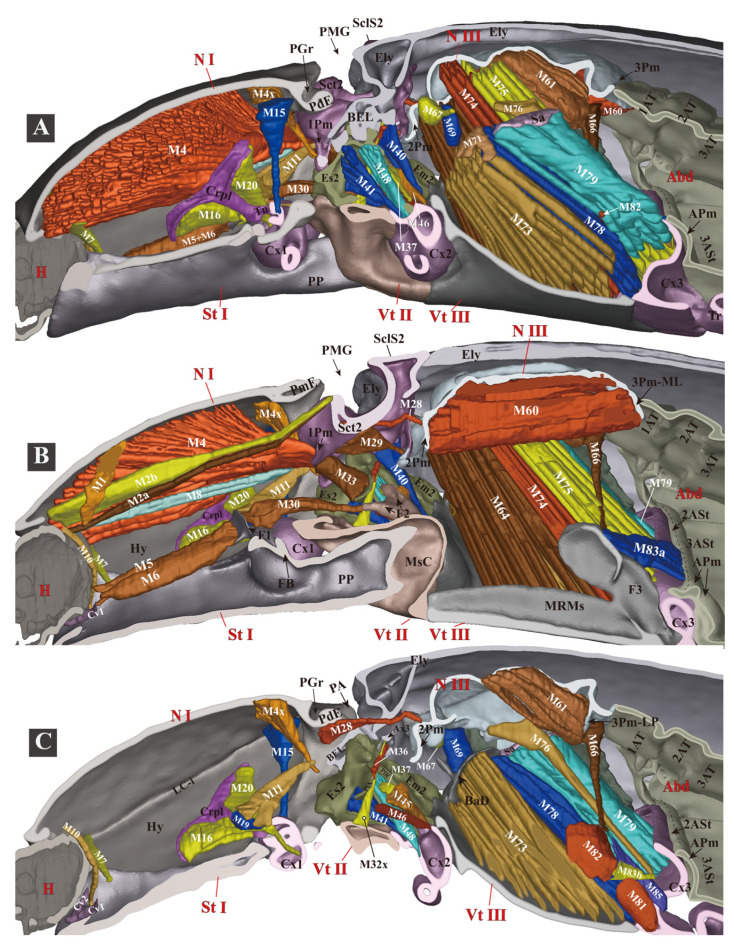
Three-dimensional reconstructions of the thoracic skeleton and musculature of *Campsosternus auratus*, lateral view. The model’s head is facing left; the exoskeleton is cut along the parasagittal and sagittal planes, to show the internal muscles. See Section 2 ‘Materials and Methods’ for technical information and abbreviations. (**A**) model is cut along the left 1/3 in the parasagittal plane. (**B**) model is cut in the sagittal plane, muscles on the left 1/2 of the body are hidden. (**C**) model is cut along the right 1/3 in the parasagittal plane, muscles on the left 2/3 of body are hidden.

**Figure 8:**
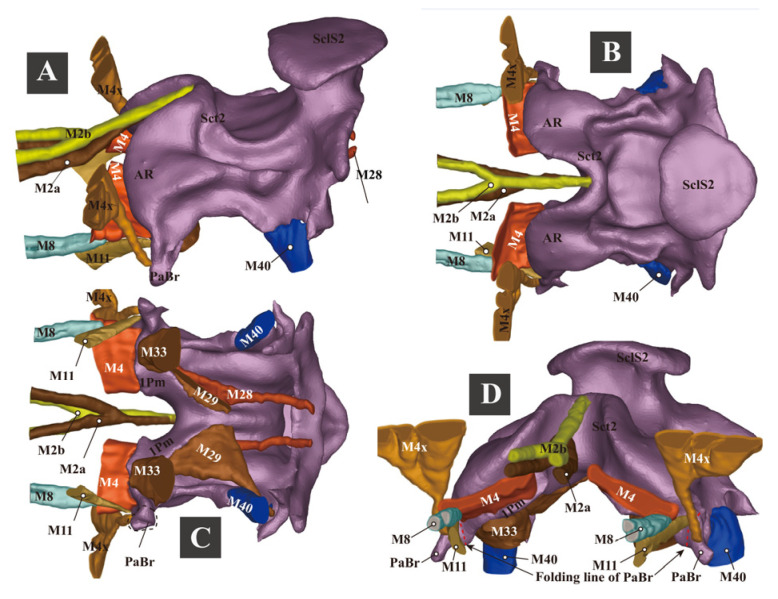
Three-dimensional reconstructions of the mesonotum and attached muscles of *Campsosternus auratus*. Muscles are partly cut to show details for other structures; see Section 2 ‘Materials and Methods’ for technical information and abbreviations. (**A**) lateral view. (**B**) dorsal view, anterior part facing left. (**C**) ventral view, anterior part facing left. (**D**) anterolateral view.

**Figure 9:**
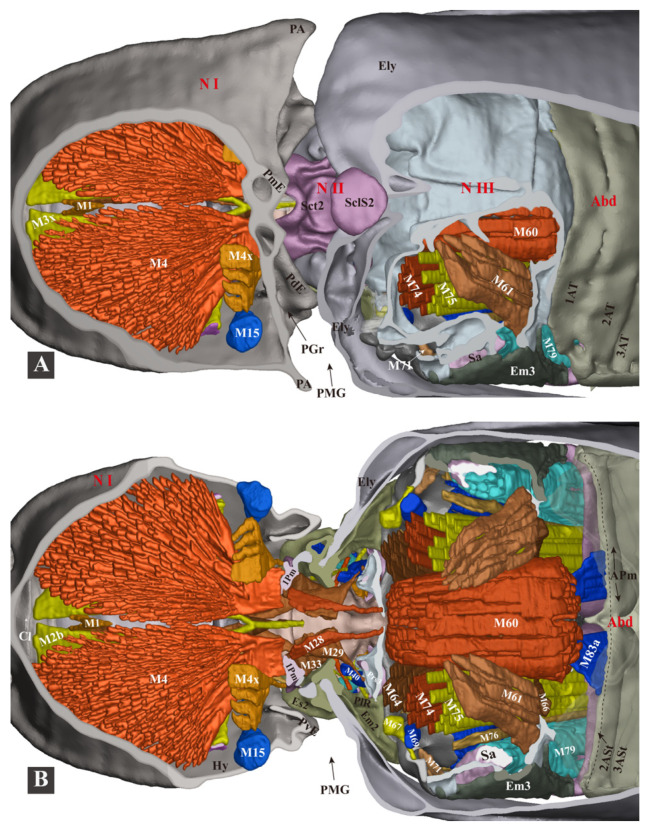
Three-dimensional reconstructions of the thoracic skeleton and musculature of *Campsosternus auratus*, dorsal view, part 1. The head of the model is facing left, and the skeleton of the model is cut in the frontal (coronal) plane at different layers (**A**,**B**) to show internal musculature; for technical information and abbreviations, see Section 2 ‘Materials and Methods’.

**Figure 10:**
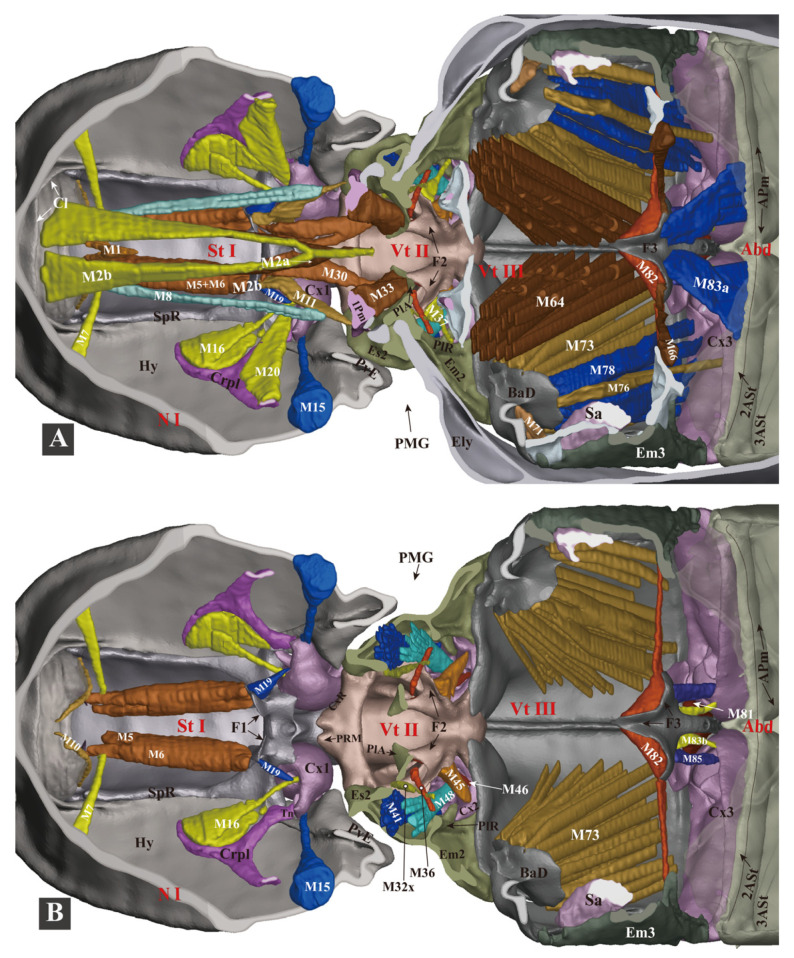
Three-dimensional reconstructions of the thoracic skeleton and musculature of *Campsosternus auratus*, dorsal view, part 2. The head of the model is facing left, and the model is cut in the frontal (coronal) plane at different layers (**A**,**B**) to show internal musculature; for technical information and abbreviations, see Section 2 ‘Materials and Methods’. Some of the muscles are removed to show the ones hidden underneath.

Beetle specimens were fixed in absolute ethanol. Hexamethyldisilane (HDMS) was used to prevent the internal contents from deforming during drying: the absolute-ethanol-preserved specimens were immersed in HDMS for 12 h and exposed to the air with ventilation for 48 h to allow the HDMS to decompose. One specimen of *C. auratus* with a resting position was scanned with a MicroXCT-400 scanner (Xradia Inc., Pleasanton, CA, USA) with the following settings: beam strength 60 kV, 8 W, and 0.4 X, absorption contrast, 360 steps, and image 1012 × 1024; the data were used for 3D reconstructions. In total, 1670 sections of images were obtained and imported to Amira 5.4.1 (Visage Imaging, San Diego, CA, USA) for segmentation; 3D structures were exported as ‘.stl’ files. These files were imported to VG Studio Max 3.0 (Volume Graphics, Heidelberg, Germany) for volume rendering; the structures were given different colors, in order to distinguish their boundaries and were cropped in sagittal or frontal planes to show internal details (Figure 3, Figure 4, Figure 5, Figure 6, Figure 7, Figure 8, Figure 9 and Figure 10; Supplementary File S2). 

Another specimen with a back-arched position was scanned for comparison purposes (not used for 3D reconstructions) using a Scanco Medical μCT100 scanner (Scanco Medical Inc., Wangen-Brüttisellen; Switzerland) with the following settings: beam strength 45 kV; 8 W, voxel size 10 μm, FOV: 20.48 mm, absorption contrast, 360 steps, image 2048 × 2048, and 4250 obtained sections. The back-arched position was made possible by freezing the live specimen at 4 °C for 20 min, to prevent it from locomotion; then, the specimen was immobilized in the back-arched position using plasticine, after which it was immediately killed using ethanol.

### 2.4. High-Speed Filming 

**Figure 11:**
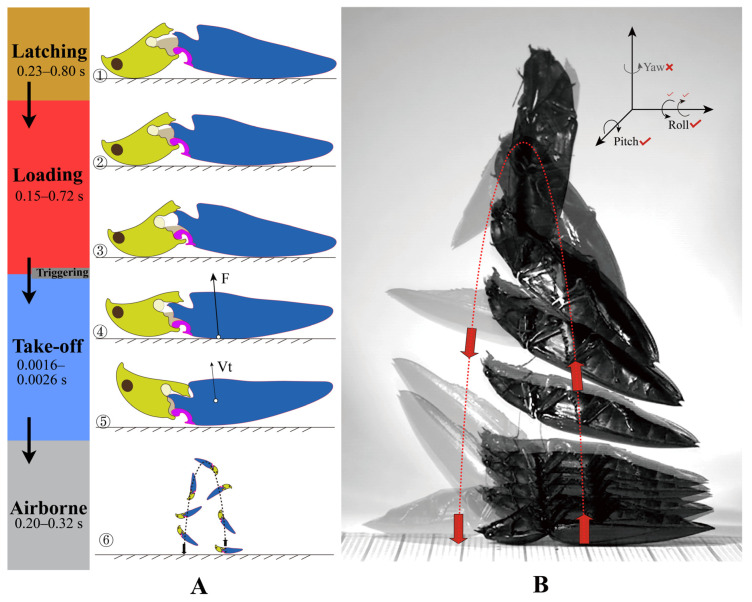
A diagram of the jumping process of *Campsosternus auratus*. ①: latching phase; ②, ③: loading phase; ④, ⑤: take-off phase; and ⑥: airborne phase. (**A**) A scheme of the jumping process: mesonotum is marked in purle; ‘F’ indicates the counterforce from the ground acting on the base of the elytra; ‘Vt’ indicates the direction of velocity at the end of the take-off phase. (**B**) The process of a typical jump: original photographs were taken using a high-speed camera, which was later superimposed into one image in Photoshop. High-speed filming parameter: 1000 fps; exposure time: 100 μs.

**Figure 12:**
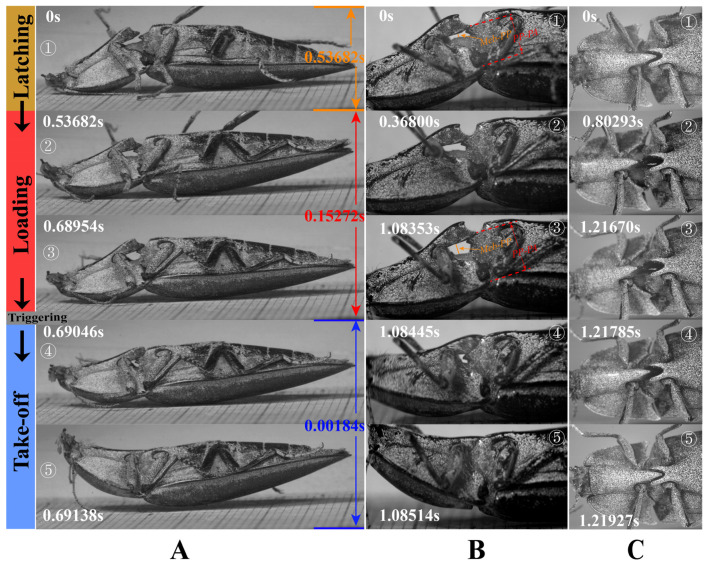
The jumping process of *Campsosternus auratus*. The airborne phase is not shown. Photos were taken using a high-speed camera at 4347.8 fps (frame interval of 230 μs) and an exposure time of 50 μs. The beetles were coated with dye penetrant inspection materials, in order to enhance the quality of high-speed filming. ①: latching phase; ②, ③: loading phase; and ④, ⑤: take-off phase. (**A**) lateral view of the body. (**B**) lateral view of the thorax; ***PP-PA*** indicates the distance between dorsal apex of prosternal process (PP) and apex of posterior angle of pronotum (PA), ***Meb-PP*** indicates the distance between Meb and ventral surface of PP. (**C**) ventral view of the thorax.

Live specimens of *C. auratus* and other elaterid species were collected in the field in various locations (see Supplementary File S1) of China from August 2020 to July 2021 for high-speed filming. During the study, beetles were reared in plastic containers in the laboratory. Containers contained barks and sawdust, and beetles were fed with insect jelly. Before the high-speed filming some individuals were coated with a white color using dye penetrant inspection materials, so that the body of the beetle was much brighter and, hence, the quality of the high-speed videos was enhanced. A layer of paper was glued closely to the surface of an acrylic plate, and the beetles were placed on this plate, inverted (i.e., with abdominal sternites facing upward). In some videos, individuals were held manually to observe their free clickings. Three LED spotlights (30–45 W) were used as the light source. Jumps of the beetles were initiated by themselves, spontaneously, or by us using a brush pen. Videos were recorded using a Revealer 5KF10MS high-speed camera (FuHuang AgileDevice Inc., Hefei, China) with Canon 100 mm and 18–135 mm lenses (Canon Inc., Tokyo, Japan), using a frame rate of 4000–8000 fps and an exposure time of 25–50 μs; a frame rate of 1000 fps was also used to analyze their jumping summersaults and jumping heights. The recorded videos were played frame-by-frame and analyzed using the auxiliary software ‘VL 3.0’ (FuHuang AgileDevice Inc., China) (Figure 11 and Figure 12). 

In total, 121 Gb of files of high-speed films was recorded for *C. auratus*, and 113 Gb was recorded for other Elateridae species for comparison purposes (see Supplementary File S1 for species information).

### 2.5. Experiment 1. Testing Functions of Essential Clicking-Related Muscles and Sclerites

We tested the functions of possible clicking-related muscles and sclerites by destroying or removing them. The results are listed in Table 2 and Table 3. This experiment was operated under a Nikon SMZ645 stereomicroscope, using a fine ophthalmic surgical microscissor, forceps, and a fine hook modified from an insect pin. Two persons were involved in the experiment: one held the beetle in position and the other cut the muscles and sclerites. 

Operation 1: M2a and M2b were cut simultaneously at their posterior insertions near the mesonotum. This operation was made possible by opening the beetle’s promesothoracic gap manually.

Operation 2: M4 was cut at its posterior insertions near the mesonotum. This operation was made possible by opening the beetle’s promesothoracic gap manually.

Operation 3: M2b was cut off at its anterior ends by removing the anteromiddle inflected margin of the pronotum.

Operation 4: M1, M2a, and M8 were cut simultaneously at their insertion near the dorsal postoccipital ridge. Operational method: the heads of click beetles were slightly pulled out of the prothorax using the forceps and fine hook, after which the muscle insertions near the postoccipital ridge were exposed and cut. 

Operation 5: M5, M6, and M7 were cut simultaneously at their insertion near the ventral postoccipital ridge; the method was similar to that of operation 4. 

Operation 6: M4x and M11 were cut simultaneously at their posterior insertions near the mesonotum. This was made possible by opening the promesothoracic gap manually. 

Operation 7: M30 was cut at its middle part. This was made possible by opening the promesothoracic gap manually. 

Operation 8: the prosternal rest of the mesoventrite (PRM) was cut at its posterior edge in the transverse plane.

Operation 9: the posterior part of the prosternal process (PP) was cut in the transverse plane (the entire friction hold was removed). 

Operation 10: the elytra were cut off, including the basal lobe of the elytron (BEL) (but the auxiliary sclerites were not removed).

Operation 11: the posterior part of the posterodorsal evagination of the pronotum (PdE) was cut in the transverse plane; its anterior bulged area (PdEB) was not removed.

Operation 12: the posterior angles of the pronotum were removed (cut in the transverse plane).

Operation 13: the head was immobilized using epoxy resin; before the resin was dry, the specimens were immobilized using plasticine.

Removing muscles sometimes resulted in the beetles not wanting to click. Therefore, stimuli such as touching or squeezing their abdomen were used in the experiment. After removing the muscles, the click beetles were stimulated to jump and observed for 5 min, then they were transferred to a plastic container and fed with insect jelly; they were kept alive for 48 h for observations. After that, they were killed and preserved in absolute ethanol. The jumping heights of some individuals were recorded based on the average of five jumps after removing the muscles.

Cutting the muscles of *C. auratus* causes hemolymph bleeding, which seems to have a minor influence on the individuals within the first few hours. However, most individuals could not survive more than 48 h.

### 2.6. Experiment 2. Observation of the Deformation of Structures in the Loading Phase 

Five individuals of *C. auratus* and two of *Sinelater perroti* (Fleutiaux) (Tetralobinae, following Kundrata et al. [27]) were observed under a Nikon SMZ645 stereomicroscope. The beetles’ anterior and posterior parts of the body were held by the left and right hands, respectively. We exerted a little force on the prothorax to prevent the prosternal process (PP) from disengaging the prosternal rest of the mesoventrite (PRM) in the loading phase. In this circumstance, the beetles would try to load the click repeatedly, and the contraction time of M4 was extended. Therefore, we had more time to observe the deformation of structures such as the prosternum, intersegmental membrane of pro- and mesothorax, and mesonotum. 

In order to measure the displacement of the prosternum in the loading phase, the high-speed filming of the lateral view of two jumps of two *C. auratus* individuals was analyzed (the frames are similar to those shown in Figure 12A,B). The distance between the dorsal apex of the prosternal process (PP) and the apex of the posterior angle of the pronotum (PA) in lateral view was measured; it is denoted as *PP-PA* (indicated in Figure 12B). Two values of *PP-PA* were measured: one is right before the loading phase, and the other is at the end of the loading phase.

The same high-speed-filming videos were used to measure the movement of the ventral surface of the pro- and mesothoracic intersegmental membrane (Figure 1G: Meb). Displacement of the Meb in the dorsoventral axis is represented by the change in the distance between the Meb and ventral surface of PP in lateral view; it is denoted as *Meb-PP* (indicated in Figure 12B). Two values of *Meb-PP* were measured: one right before the loading phase, and the other at the end of the loading phase. Displacement of the Meb in the longitudinal axis is represented by the travel distance of a point at the middle part of the Meb in lateral view. 

### 2.7. Recording of the Clicking Sounds

Human fingers held the specimens in the air to prevent them from hitting the ground, which may cause potential noise in the recording. The same method was also used in the high-speed filming. The sound of three clicks of three individuals was recorded. Their clicking motions were recorded by high-speed filming, based on which the oscillation frequency of the back and forth movement of the anterior and posterior parts of the body was calculated. A Sony ICD-PX240 recording pen (Sony Inc., Tokyo, Japan) was used to record the sound. The audio files were imported into GOLDWAVE software (GoldWave Inc., St. John’s, NL, Canada). The frequency of the sound was calculated based on an oscillogram generated in the GOLDWAVE software (Figure 14).

## 3. Results

### 3.1. The Functional Morphology of the Thorax of Campsosternus auratus

#### 3.1.1. General Morphology

The body is ovoid and elongate, gradually narrowing from the middle to the anterior and posterior ends in dorsal view; it is flattened dorsoventrally in the lateral view (Figure 1 and Figure 3; Supplementary File S2). The exoskeleton is strongly sclerotized. The body surface is smooth and metallic green, with minute round punctures and a golden-to-purple luster. The ventral surface of the body bears soft and fine setae. Bodyweight of live individuals: 1.09–1.96 g (n = 10); body length: 32.0–45.0 cm (n = 10); and body width: 1.1–1.4 cm (n = 10). Similar to many other (typical) click beetles, the thorax of *C. auratus* has a robust and elongate prosternal process, a deeply concaved mesoventral cavity, well-developed pronotal posterior angles, a specialized mesonotum, and a strongly sclerotized elytral base. Its thorax is strongly sclerotized; the posterior part of the prothorax is fitted with the anterior part of the mesothorax, both of which are responsible for the clicking and form the promesothoracic interlocking mechanism.

#### 3.1.2. Head

The head is flattened dorsoventrally, ovoid. The posterior part (postocciput) is connected with the prothorax by the cervical membranes. The following muscles are connected to the posterior part of the head: M1, M2b, M5, M6, M7, M8, and M10. **Functions.** Evans [13] found that, in several cases of high-speed filming of the jump of *Athous haemorrhoidalis*, the head moved slightly forwards (protruded out of the thorax) just before the jump. He hypothesized that the protraction of the head could be the trigger of the clicking. Based on our study, protraction of the head presents in the clicks of *C. auratus*. However, the clicking mechanism of *C. auratus* does not rely on protraction of the head, as the immobilization of the head in the retracted position does not affect the clicking mechanism (see Table 3). Furthermore, in the clicking of *Sinelater perroti*, protraction of the head was absent (four jumps of one individual were filmed and observed).

**Cervical sclerites** (Cv, Figure 4I). Cv consists of two sclerites: Cv1 (anterior cervical sclerite) and Cv2 (posterior cervical sclerite), both of which are weakly sclerotized, flat as well as elongated, and deeply embedded in the cervical membrane connected to the head. Cv1 and Cv2 are connected by elastic cuticles at their anterior ends. Cv2 is on the posterolateral side of Cv1. **Functions** (of the Cv and cervical membranes). The movements of the head.

#### 3.1.3. Prothorax

(1) **Pronotum** (Figure 4D)

The pronotum (N I) is subquadrate in dorsal view; ovoid and slightly flattened in lateral view. The lateral carina and hypomeron of the pronotum are well-developed. The pronotum has well-developed posterior angles (PA), a common characteristic in Elateridae. In the ventral view the anterior margin of the pronotum is well-inflected posteriorly and internally, forming a well-sclerotized collar (Cl). 

**Posteromedian part of the pronotum** (PmPr). The PmPr slightly expands posteriorly, with a concave and extremely smooth ventral surface. **Functions**. The concave ventral surface of the PmPr accommodates the raised anteromedian area of the mesonotum during the clicking process. 

**Posterior evaginations of the pronotum** (PE). PE are the evaginated parts of the posterior margin of the pronotum, which can be divided into posterodorsal evagination (PdE) and posteroventral evagination (PvE). PE are well-sclerotized and have a highly smooth surface, forming a socket-shaped structure at the posterior end of the prothorax. 

**Figure 13:**
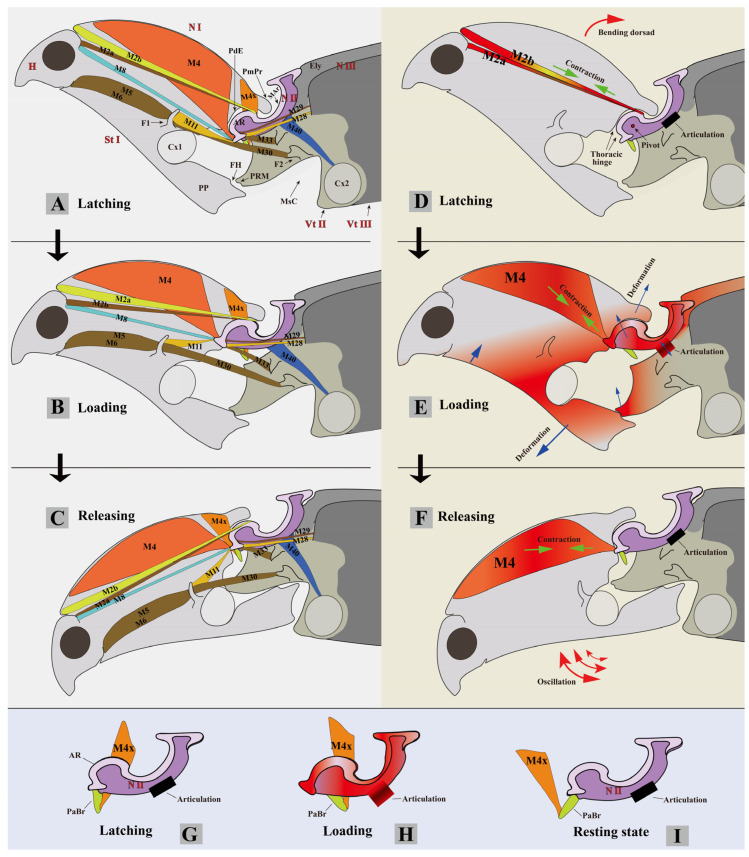
A diagram of the clicking mechanism and the deformation of thoracic structures in *Campsosternus auratus*. (**A**–**C**) show the major skeleton and muscles in the pro- and mesothorax. (**D**–**F**) show the contraction of muscles and the deformation of thoracic structures in the clicking process; the tension in the thoracic structures is indicated with a red gradient color; blue arrows show the deformation of skeleton; green arrows show the contraction of M2 and M4; and black and red rectangles indicate the articulation between the mesonotum and mesopleuron. (**A**,**D**) latching phase: red arrow shows the back-arching movement of the prothorax when muscles M2a and M2b contract. (**B**,**E**) loading phase: the enormous M4 contracts, to load the biological springs. (**C**,**F**) releasing phase: biological springs release the elastic energy stored in the previous phase. (**G**–**I**) the shape of the mesonotum in different stages (latching, loading, and resting): the mesonotum is in its original form in the resting and latching phases, and bent dorsad in the loading phase. (**G**–**I**) also show that the prealar bridge is significantly pulled posterad in the loading phase when M4x contracts.

**Posterodorsal evagination** (PdE). The PdE is the evaginated part of the posterodorsal margin of the pronotum, with a highly smooth ventral and posterior surface. The anterior part of the PdE has a prominently bulged area (PdEB), also called the ‘knob’ by Evans [13]. **Functions**. The PdE is one of the essential structures involved in the clicking mechanism. The PdE and the anterolateral region of the mesonotum (AR) constitute the ‘thoracic hinge’ (defined in this study, see Figure 13D), which plays important roles in the latching, loading, and take-off phases. The ‘pivot’ (Figure 13D) is situated in the center of the AR in the clicking mechanism. In the loading phase of the clicking mechanism, when M4 contracts, the pronotum moves slightly posteriorly and the posterior part of the PdE is pushed onto the basal lobes of the elytron (BEL); this prevents the pronotum from moving further backward, allowing for the building-up of elastic energy in the thorax. When the PdE was removed (with the anterior bulged area retained) the loading motion was significantly weakened and the clicking mechanism was disrupted (see Table 3). According to Evans [13], in the clicking process, the bulged area (PdEB) of the PdE slides into the lateral groove of the mesonotum (LGr, i.e., mesonotal grooves), and together they form the ‘pivot’. However, based on our dissections and observations in *C. auratus*, the PdEB did not slide into the LGr during the clicking or promesothoracic interlocking mechanism. The PdEB in *C. auratus* provides constraints to establish the thoracic hinge, and it may also help prevent the pronotum from moving further backward when M4 contracts.

**Posteroventral evagination** (PvE). The PvE is the evaginated part of the posterior margin of the hypomeron. It is strongly sclerotized and has a highly smooth internal surface. **Functions**. When the pro- and mesothorax are interlocked the PvE is pushed and tightly locked onto the anterior evagination of the mesanepisternum. 

**Posterodorsal groove** (PGr). The PGr is situated above the pronotal posterodorsal evagination (PdE). **Functions**. The PGr fits with and accommodates the flange of the elytral base (EBF) when the pro- and mesothorax are interlocked.

**Posterior angle of the prothorax** (PA). The PA is wedge-shaped, well-sclerotized, and produces posterolaterally. **Functions.** The PA fits with the basolateral part of the elytra when the thorax is interlocked. In this study the fingers of two of our colleagues were accidentally injured by the clamping of the pro- and mesothorax of *Sinelater perroti* (Tetralobinae). Indeed, as the PA is acute it may potentially injure natural enemies when the thorax is interlocked. The removal of the PA does not affect the clicking mechanism (see Table 3).

(2) **Cryptopleuron** (Figure 4J)

The propleuron is invisible externally and reduced to the cryptopleuron (Figure 4: Crpl), which is T-shaped, with M16 and M20 attached. The distal part of the Crpl is partly merged with the trochantin. **Functions.** The cryptopleuron is responsible for the rotary movement of the procoxa and is not directly involved in the clicking mechanism.

(3) **Prosternum** (Figure 4F–H)

**Prosternal process** (PP). The PP is well-sclerotized, and resembles a wedge. In lateral view the PP has a concave posterior end (i.e., the area between the ventral and dorsal apices (sensu [41,42]); the dorsal apex is much more strongly produced posteriorly than the ventral apex. **Functions**. The PP plays an essential role in the clicking mechanism, especially in storing, loading, and releasing elastic energy. The posterodorsal part of the PP is latched onto the prosternal rest of the mesoventrite (PRM) in the latching phase, so that the following loading phase is possible. In the loading phase the PP is deformed, so that elastic energy can be stored. In the take-off phase the PP disengages from the PRM; then, the dorsal surface of the PP slides on the ventral surface of the mesoventral cavity (MsC) at high speed. In this way, the PP plunges into the MsC abruptly. The specific morphology of the posterior part of the MsC may play an essential role in slowing down the PP when it plunges into the MsC. Removing the posterior part of the PP (including the friction hold) resulted in the clicking mechanism being disrupted, because the PP and PRM were unable to establish the correct latching position; the PP was stuck on the PRM when the beetle tried to click (tested in Experiment 1, see Table 3).

**The friction hold** (FH). The FH is a coarse region covered with a velvet-like hair cushion situated at the posterior fourth of the dorsal surface of the prosternal process. The FH is a lower terrace, and its anterior part is a higher step. The rest part of the dorsal surface of the prosternal process is higher than the FH and is exceptionally smooth. According to Evans [13], in *Athous haemorrhoidalis* the dorsal surface of the FH is coarse, with minute parallel ridges without a hair cushion, which is very different from *C. auratus*. Therefore, the FH may also have variable surface textures in other species. **Functions**. The FH and the prosternal rest of the mesoventrite (PRM) are the critical structures that constitute the trigger system. They enable the latching, loading, and triggering processes. In the latching phase the anterior part of the FH (i.e., the area near the step) is set onto and latched with the PRM. They lock the entire system temporarily, so that the thoracic structures can deform and store elastic energy when M4 contracts. When the increase in the elastic energy exceeds the tolerance of the locking between the FH and PRM they disengage from each other, allowing the entire system to release the elastic energy and translate it into kinetic energy. 

**Profurca** (F1). The F1 is fan-shaped, with M5, M6, M30, and M11 attached. The furcal pit is visible in the ventral view when the procoxa is removed. The profurcal base (FB) is well-sclerotized, with a bulge that Evans described as a ‘bumper’ (the bulge is absent in the *Phorocardius unguicularis* examined in this study). Under the bulge there is an intercoxal wall between the two coxae, which is ridge-shaped in the caudal view. **Functions**. When the pro- and mesothorax are interlocked tightly, the V-shaped prosternal rest of the mesoventrite (PRM) is stuck on the ridge-shaped intercoxal wall, and the dorsum of the PRM is also constrained by the basal bulge of the furca. 

**Pronotosternal suture** (PsS). The anterior part of the PsS is flexible; the posterior part is more closely attached to the hypomeron by a strong elastic cuticle, and a pronotosternal articulation (Figure 1F and Figure 4A: PSA) is present at the posterior end of the PsS. The PSA consists of a socket on the wall of the hypomeron and a knob-shaped process on the prosternum, which interlock with each other closely. **Functions**. In the loading phase, the prosternal process is pushed ventrally; as the fulcrum is situated at the PSA, the anterior part of the prosternum is levered dorsally. The flexible anterior part of the PsS may have the advantage of storing elastic energy in the loading phase, and it may also contribute to the disengagement of the friction hold (FH) and the prosternal rest of the mesoventrite (PRM) at the beginning of the take-off phase. In *Sinelater perroti* the pronotosternal suture is entirely sclerotized, and the entire prosternum is fused with the hypomeron. This species jumps far less explosively than other species; based on six jumps by two individuals, their jumping height (displacement of the center of mass) barely exceeds their body length. 

**Procoxa** (Figure 4: Cx1). The procoxa is spherical. The procoxa is not directly involved in the clicking mechanism. 

#### 3.1.4. Mesothorax

(1) **Mesonotum** (Figure 1, Figure 2, Figure 5, Figure 8 and Figure 13: N II)

The **mesonotum** (N II) is specialized into a saddle-shaped structure and strongly sclerotized. It consists of the mesoscutum (Sct2) anteriorly and the mesoscutellum (Scl2) posteriorly; the scutoscutellar suture between them is indistinct. In lateral view, the mesonotum is sinusoidal or saddle-shaped, with the middle part strongly concave and arched ventrally (MAr), with the anterior and posterior regions raised dorsally. In dorsal view, lateral grooves (LGr) are present at the lateral sides of the median-arched area. In lateral view, the anterior part of the mesonotum is free, and the middle lateral part of the mesonotum is tightly articulated with the elytral axillaries and mesopleuron by strong elastic cuticles. The tight articulation provides a strong constraint for the mesonotum. The posterodorsal part of the mesonotum is not articulated with any sclerites; it forms the elytral-mesoscutellar, interlocking (see the following text for more information) with the base of the elytra. The posteroventral margin of the mesonotum is loosely connected with the prescutum of the metanotum (Prs3) by a very flexible intersegmental membrane. The yoke plate (YP) of the mesonotum has a tight connection with the metathoracic prescutum (Prs3). **Functions.** The anterior part of the mesonotum is the thoracic hinge and rotation center of the clicking system (see Figure 13E); it is also biologically specialized for the storage and abrupt release of elastic energy. In the loading phase the anterior part of the mesonotum is slightly deformed and pulled dorsad towards the pronotum by the contraction of M4 (Figure 13E). This was supported by ‘Experiment 2’ (for more details, see Section 2 Materials and Methods). It revealed that the mesonotum and intersegmental membrane of the pro- and mesothorax are lifted dorsally during the loading phase, and that their shape is restored when the loading motion is ended. The mesoscutellar shield inclines backward and is pushed onto the base of the elytra in the loading phase, and it restores the original position when the loading motion is ended. The displacement of the mesoscutellar shield also confirms the deformation of the anterior part of the mesonotum. We hypothesize that the sophisticated saddle-like shape of the mesonotum may have an advantage for the storage and quick release of elastic energy.

**Mesoscutellar catch** [13]. Equivalent to the **elytral-mesoscutellar interlocking** [43], see the following text for more information. 

**Anteromedian emargination of the mesonotum** (Figure 5A: AmE). The AmE is defined in this study: it is a spherical emargination at the anteromedian part of the mesonotum. M2 (M2a + M2b) goes through the AmE and is attached to the raised anterodorsal part of the mesonotum. **Functions**. The AmE makes room for the insertion of M2. These two muscles provide a torque, driving the prothorax rotating dorsad (i.e., back-arching movement, see Figure 13D).

**Anterolateral region of the mesonotum** (AR, Figure 1I and Figure 5A). The AR is defined in this study. It is the oblique and somewhat hemispherical area at each side of the anterior part of the mesonotum, and it has a highly smooth surface. Its dorsal surface fits with the posterodorsal evagination (PdE). Its anterior part adjoins the inflected anterior margin of the mesonotum (Figure 5: IAM, defined in this study). **Functions.** In the latching phase, the PdE is pushed onto the AR by M4, and they form the thoracic hinge and rotation center for the entire clicking mechanism; the pivot is situated in the center of the AR (see Figure 13D). The AR plays the role of a condyle, while the PdE is a socket.

**Prealar bridge of the mesonotum** (PaBr). The PaBr is a triangular, well-sclerotized sclerite. M4x is attached to the PaBr. The PaBr is attached to the anterolateral side of the mesoscutum by an elastic cuticle. **Functions**. The junction between the PaBr and the mesoscutum plays a role as the ‘folding line’ (Figure 5A,D) of the PaBr. In the latching phase, when the prothorax is bent dorsad (back-arched), the PaBr is pulled by M4x and folded posteriorly along the ‘folding line’. The displacement of the PaBr causes the change in the direction of M4x. In the loading phase M4x contracts and, more significantly, pulls the PaBr posterad. We hypothesize that these phenomena result in the PdE being pressed on the AR more intensely. By these means, the thoracic hinge may be strengthened, and the clicking performance may be improved. The displacement of the PaBr is present in the clicking mechanism of both *C. auratus* and *Sinelater perroti*.

**First phragma** (1Pm). The 1Pm is strongly developed, with a specialized complex shape, and situated at the anteroventral part of the mesonotum; it is separated from the inflected anterior margin of the mesonotum (IAM) by the dorsal part of the intersegmental membrane of the pro- and mesothorax (Figure 1G and 6B: Meb). M4 is inserted on the anterior part of the 1Pm. M8 and M11 are inserted on the anterolateral part of the 1Pm. 

**Median-arched area of the mesonotum** (MAr). The MAr is strongly concave and arched ventrally, with the anterior and posterior parts strongly raised dorsally. **Functions**. The MAr accommodates the posteromedian part of the pronotum (PmPr) when the prothorax is bent dorsad in the latching phase. We hypothesize that the arc-like shape of the MAr makes it easier for the mesonotum to be deformed, store, and release elastic energy. 

**Mesoscutellum** (Scl2). The mesoscutellar shield (SclS2) is significantly raised above the surface of the mesonotum. The mesoscutellar shield and lower part of the mesoscutellum can fit with the mesal part of the elytra and form the elytral-mesoscutellar interlocking (see the following text for more information). The elytral-mesoscutellar interlocking may constrain the posterior part of the mesonotum and help the mesonotum sustain the force generated by M4 in the loading phase. 

**Yoke plate** (YP, [29]). The YP is a ventrad producing area at the posterolateral part of the mesonotum, which is closely connected with the metathoracic prescutum (Prs3) by the elastic cuticle.

(2) **Elytron** (Figure 5: Ely)

The elytron is strongly sclerotized, elongated, and gradually narrowing, from the base to the pointed apex, with a smooth dorsal surface. The base of the elytra and the posterior part of the mesonotum form the elytral-mesoscutellar interlocking. Furthermore, two elytra interlock with each other at the elytral suture. **Functions**. The elytron is essential for the clicking mechanism. In *C. auratus*, the removal of the elytra resulted in the loss of clicking ability. First, removing the elytra caused the loss of the elytral–mesoscutellar interlocking, so the mesonotum produced excessive displacement when M4 contracted. Second, in the loading phase the posterior evagination of the pronotum (PdE) also lost constraint when the basal lobe of the elytron (BEL) was absent, so the pronotum moved much further backward. These abnormalities finally caused the friction hold (FH) to be unable to properly lock onto the prosternal rest of the mesoventrite (PRM). When the elytron (either one) was removed, the prosternal process was dislocated during the loading phase, and the clicking mechanism was disrupted (see Table 3).

**Basal lobe of the elytron** (BEL). The BEL is robust and strongly sclerotized. It contains the humeral plate (HP, i.e., the base of the costa), the base of the subcostal (BSc), and the base of the radius vein. **Functions**. The BEL works as a ‘stop block’, contributing to the constraining of the pronotum. In the latching and loading phases, the posterodorsal evagination of the pronotum (PdE) is set on the anterolateral region of the mesonotum (AR). When M4 contracts the pronotum tends to slide posteriorly, but the BEL encounters the PdE and stops the pronotum from moving further backward. By these means, the BEL contributes to establishing the thoracic hinge system for the clicking mechanism. 

**Basal elytral groove** (BEG) [13]. The BEG is a prominent groove situated at the base of the elytra. The posterodorsal evagination of the pronotum (PdE) is accommodated in the BEG when the pro- and mesothorax are interlocked. 

**Flange of the elytral base** (EBF) [13]. The EBF is situated above the basal elytral groove (BEG). When the pro- and mesothorax are interlocked the EBF is inserted into the posterodorsal groove (PGr) of the pronotum. 

**Elytral-mesoscutellar interlocking** [38,43] (also known as the mesoscutellar catch [13]). The mesal process of the elytral base (Figure 5N,O: EBP) is adapted to the dorsal surface of the mesoscutellum (under the anterior part of the mesoscutellar shield); the proximal part of the mesal rim of the elytra fits into and becomes stuck on the ventral side of the posterior part of the mesoscutellar shield. By these means, the elytral base and the mesonotum form the elytral-mesoscutellar interlocking. **Functions**. The elytral-mesoscutellar interlocking is essential for the clicking mechanism. On the one hand, it prevents the opening of the elytra [43]. On the other, it constrains the posterior part of the mesonotum, so that when M4 contracts the mesonotum can sustain the deformation and store a colossal amount of elastic energy for the clicking mechanism. Without the elytral-mesoscutellar interlocking, the mesonotum would produce excessive displacement when M4 contracts.

(3) **Mesopleuron** (Figure 5: Es2, Em2)

The mesopleuron consists of the mesanepisternum (Es2) and mesepimeron (Em2), which are closely fused. They are delimited by the mesopleural suture (PlS) externally and the pleural ridge (PlR) internally. The mesopleuron is closely articulated with the mesoventrite and metaventrite by elastic cuticles. 

**The mesopleural wing process** (PlWP2). The PlWP2 is situated at the dorsal part of the pleural ridge, whose lateral part is articulated with the middle lateral part of the mesonotum, and dorsal part is articulated with the elytral axillaries. These together form a strong articulation.

**Mesanepisternum** (Es2). The Es2 has a prominent anterior evagination, with a highly smooth surface. The anterior evagination of Es2 forms a conformal contact with the posteroventral evagination of pronotum (PvE), and they contribute to the promesothoracic interlocking mechanism. 

**Mesepimeron** (Em2). The dorsal edge of the Em2 fits with the epipleuron of the elytron, and their conformal contact may contribute to strengthening the stability of the mesothorax.

(4) **Mesoventrite** (Figure 5H–K: Vt II)

The mesoventrite is deeply excavated in the ventral view; the prosternal rest of the mesoventrite (PRM) produces anteriorly, with a V-shaped incision at its anterior edge. The anterolateral part of the mesoventrite forms the procoxal rests (CxR) to accommodate the posterior part of the procoxae. The posterior margin of the mesoventrite is entirely fused with the metaventrite, and they are delimited by a weak line (sensu the ‘mesometaventral junction’ by Lawrence et al. [36]). In lateral view, the posterior part of the mesoventrite forms acetabula (Act) to accommodate the mesocoxae; the posterodorsal part of the mesoventrite bears the mesothoracic furca, whose arms are well-developed. In dorsal view of the mesoventrite, the anterior and lateral margins are strongly inflected, and the lateral area is deeply concave. 

**Prosternal rest of the mesoventrite** (PRM). The PRM is situated at the anterior part of the mesoventrite. The PRM is incised at its anterior margin and V-shaped. Its surface is highly smooth. **Functions.** The PRM and prosternal process (PP) are the most critical structures that constitute the trigger system of the jumping mechanism. In the latching phase they latch onto each other and establish a locking position, making the following loading phase and triggering process possible. The PRM also bears enormous stress during the clicking process. The removal of the PRM resulted in the clicking mechanism being disrupted, because the PP and PRM were unable to establish the correct latching position, and the PP was stuck on the anterior cut part of the mesoventrite when the beetle tried to click (tested in ‘Experiment 1’, see Table 3). 

**Mesoventral cavity** (MsC). The MsC is cuneiform. The lateral walls of the MsC have a coarse surface. The ventral wall is extremely smooth and slightly concave along the middle, each side with a slightly raised longitudinal ‘track’ (sensu [13]), along which the prosternal process slides. 

#### 3.1.5. Metathorax 

The metathorax (Figure 6) is not directly involved in the clicking or the promesothoracic interlocking mechanism. Its general form is not dramatically different from that of other beetles.

(1) **Metanotum** (N III)

The metanotum is very weakly sclerotized. It is in contact with the mesonotum: the median part of their border is loosely connected with a flexible membrane; at their lateral sides the metathoracic prescutum (Prs3) is closely attached to the mesonotal yoke plate by an inflexible elastic cuticle. The lateral and posterior borders of the metanotum are loosely connected by flexible membranes with the metapleuron and abdominal tergite, respectively. Its anterolateral area bears the axillary sclerite and hind wing.

(2) **Metapleuron** (Pl III)

The metapleuron consists of the metanepisternum (Es3) and metepimeron (Em3). The Es3 is well-sclerotized and somewhat wedge-shaped, with the anterodorsal part strongly produced, bearing the knob-like metathoracic pleural wing process (PlWP3) and basalar sclerite (Ba). In the internal view, the Ba is strongly developed and produced posteroventrally, forming an enormous basalar disc at its posterior end. The Em3 is weakly sclerotized and elongated; the dorsal part slightly turns towards the metanotum, and a flexible membrane connects them.

(3) **Metaventrite** (Vt III)

The metaventrite (Vt III) is strongly sclerotized, with a generally quadrate shape. In ventral view, the anterior margin (including the anterior margin of the metaventral process (MtP) and the metaventral acetabula (Act)) are entirely fused with the mesoventrite, leaving a faint trace. The anterolateral margin is closely articulated with the mesepimeron (Es2); the lateral margin is articulated with the metanepisternum (Es3). A trace of discrimen is present along the middle. The middle part of the posterior margin is angulated and produced posteriorly, with a longitudinal concave furcal pit (PF) at the middle. The median ridge is strongly developed and well-sclerotized. The metafurca (F3, i.e., metaendosternite) is well-developed and cruciform.

The metacoxa is strongly transverse, with a well-developed coxal plate; the anteromedial part of the coxal rim bears a prominent internal trochantinal disc. 

#### 3.1.6. Thoracic Musculature

We follow the muscle nomenclature (**M1** to **M85**) by Larsén [16]; the muscle nomenclature used by Friedrich and Beutel [44] is put in parentheses; and **O** and **I** are abbreviations for the origin and insertion of muscle, respectively.

(1) **Prothoracic Musculature** (Figure 7, Figure 8, Figure 9 and Figure 10)

**M1** (Idlm2): *M. pronoti primus*. **O:** anteromedian part of the pronotum; **I:** dorsolateral part of the postoccipital ridge. Slender, flat, and Y-shaped. **Functions**. Movements of the head; removing M1 does not disrupt the clicking mechanism (see Table 2).

**M2a** (Idlm1): *M. pronoti secundus*. **O:** median dorsal apex of the first phragma (1Pm); **I**: dorsolateral part of the postoccipital ridge. Long, slender, Y-shaped, and underneath M2b. **Functions**. M2a contracts to retract the head, pull the prothorax closer to the mesothorax, and, more importantly, bend the prothorax dorsally (back-arching movement). M2a is present in most Coleoptera studied previously (based on the data in [16]). Removing M2a + M2b results in the loss of the clicking mechanism (see Table 2).

**M2b** (Idlm1): *M. pronoti secundus*. **O:** median dorsal apex of the first phragma (1Pm); **I:** collar of the pronotum (inflected anterior margin of the pronotum). Long, robust, Y-shaped, and situated on the dorsal side of M2a. M2b was not mentioned by Larsén [16]; it is unknown if it is present in other Coleoptera groups. **Functions**. M2b is involved in the latching phase of the clicking mechanism; contraction of the muscle results in the prothorax being pulled closer to the mesothorax, and, more importantly, being bent dorsally. Removing M2a + M2b results in the loss of the clicking mechanism (see Table 2).

**Functions of M2 (i.e., M2a + M2b)**. In lateral view the insertions M2a and M2b are situated on the dorsal side of the pivot of the thoracic hinge (Figure 13D); the contraction provides a torque, driving the prothorax to rotate dorsally around the thoracic hinge. This is referred to as the ‘back arching’ [13] movement of the prothorax. M2a and M2b are antagonistic muscles to M4 [13]. They are attached to the dorsal part of the mesoscutum, while M4 is attached to the ventral part of the mesoscutum. The contraction of M2a and M2b resulted in the rotation of the prothorax dorsad (i.e., back-arching movement) in the latching phase, while the contraction of M4 resulted in the bending of the prothorax ventrad (i.e., jack-knifing movement) in the loading and take-off phases. Removing either M2a or M2b does not affect the clicking mechanism; however, removing M2a and M2b simultaneously results in the loss of the clicking mechanism, because the click beetles are unable to bend the prothorax dorsad. When the thorax was pushed dorsally by humans they could perform a single click (see Table 2). 

**M4** (Idlm5): *M. pronoti quartus*. **O:** major area of the pronotum; **I:** anterolateral part of the first phragma (1Pm). Strongly developed. **Functions** (see Figure 13). M4 is the largest pronotal depressor muscle and the most critical muscle that functions in the clicking mechanism, occupying almost half of the volume of the prothorax. Based on the voxel data exported from Amira software, M4 is the largest muscle (1,357,799 voxels) in the body, followed by M60 (841,418 voxels) and M75 (836,675 voxels). The high level of development of M4 enables *C. auratus* to acquire sufficient energy for the clicking and thoracic interlocking behaviors. The contraction of M4 pulls the pronotum closer to the mesonotum. In the clicking process, M4 provides essential energy for the loading phase and drives the abrupt ventrad bending movement of the prothorax (sensu jack-knifing [13]) in the take-off phase; M4 also deforms the mesonotum by pulling its anterior part dorsad (see Figure 13E). In *Sinelater perroti*, the contraction of M4 led to a powerful clamp of the pro- and mesothorax, and caused injury to human fingers placed in the promesothoracic gap. 

**M4x** (Idlm5). **O:** posterior part of the pronotum (N I); **I:** prealar bridge of the mesonotum (PaBr). M4x was not mentioned for Elateridae by Larsén [16]. It is much weaker than M4, and fan-shaped. **Functions**. In the loading phase, M4x contracts and significantly pulls the prealar bridge (PaBr) posterad. We hypothesize that the contraction of M4x may strengthen the thoracic hinge by pressing the posterodorsal evagination of the pronotum (PdE) onto the anterolateral region of the mesonotum (AR). Although the removal of M4x does not result in the loss of the clicking mechanism (see Table 2), M4x may improve the clicking performance. 

**M5** (Ivlm3): *M. prosterni primus*. **O:** profurcal arm (F1); **I:** posterior tentorial arm of the head. Moderately developed, longitudinal. **Functions**. Retraction of the head. Removing M5 does not result in the loss of the clicking mechanism (see Table 2).

**M6** (Ivlm1): *M. prosterni secundus*. **O:** profurcal arm (F1); **I:** ventral part of the cervical membrane and the anterior cervical sclerite (Cv1). Moderately developed, longitudinal. **Functions**. Movements of the head; removing M6 does not result in the loss of the clicking mechanism (see Table 2).

**M7** (Idvm6): *M. dorsoventralis primus*. **O:** anterolateral region of the pronotum (N I); **I:** ventrolateral part of the cervical membrane. Slender, flat, and longitudinal. **Functions**. Movements of the head; removing M7 does not result in the loss of the clicking mechanism (see Table 2).

**M8** (Idvm8): *M. dorsoventralis secundus.* **O:** lateral part of the first phragma (1Pm); **I:** dorsolateral part of the postoccipital ridge. Slender, longitudinal. **Functions**. Movements of the head; removing M8 does not affect the clicking mechanism (see Table 2). 

**M10** (Idvm2, 3): *M. dorsoventralis quartus*. **O:** anterolateral part of prosternum (St I); **I:** dorsal part of the postoccipital ridge. **Functions**. Movements of the head; not involved in the clicking mechanism.

**M11** (Idvm10): *M. dorsoventralis quintus*. **O:** profurcal arm (F1), **I:** lateral part of the first phragma (1Pm). Slender, flat, and somewhat fan-shaped. **Functions**. The contraction of M11 pulls the prothorax closer to the mesothorax. It is unknown if it helps to latch the prosternal friction hold (FH) onto the prosternal rest of the mesoventrite (PRM) in the latching and loading phases. Removing M11 does not result in the loss of the clicking mechanism (see Table 2), but it is not certain if M11 improves the clicking performance.

Notes: M12 (*M. noto-pleuralis*) is absent in *C. auratus* (five ethanol-preserved individuals were dissected to confirm this). However, according to Larsén [16], M12 is present in *Selatosomus aeneus* (Elateridae) and many other Polyphaga species. 

**M15** (Idvm16, 17): *M. noto-coxalis*. **O:** posterolateral part of the pronotum (N I); **I:** process of the procoxa (Cx1). Moderately developed, conical. Not involved in the clicking mechanism.

**M16** (Ipcm4): *M. episterno-coxalis*. **O:** anterior part of the cryptopleuron (Crpl); **I:** process and rim of the procoxa (Cx1). Moderately developed, fan-shaped, and divided into two bundles. Not involved in the clicking mechanism.

**M19** (Iscm2): *M. furca-coxalis*. **O:** profurcal arm (F1); **I:** process of the procoxa (Cx1). Weak and small, fan-shaped, and divided into two bundles. Not involved in the clicking mechanism.

**M20** (Ipcm8): *M. pleura-trochanteralis*. **O:** posterior part of the cryptopleuron (Crpl); **I:** trochanter of the proleg. Moderately developed, fan-shaped. Not involved in the clicking mechanism.

(2) **Mesothoracic Musculature** (Figure 7, Figure 8, Figure 9 and Figure 10)

**M28** (IIdlm1): *M. mesonoti primus*. **O:** posterior part of the first phragma (1Pm); **I:** dorsal part of the second phragma (2Pm). Weak, slightly fan-shaped. M28 is attached to the clicking-related structure (mesonotum), but it is unknown if M28 is involved in the clicking mechanism.

**M29** (IIdlm2): *M. mesonoti secundus*. **O:** posterior part of the first phragma (1Pm); **I:** anterolateral part of the metathoracic prescutum (Prs3). Moderately developed, fan-shaped. **Functions**. M29 and M33 open the elytra by turning the mesoscutellum forwards [45]. It is unknown if M29 is involved in the clicking mechanism.

**M30** (Ivlm7): *M. mesosterni primus*. **O:** proforcal arm (F1); **I:** mesofurcal arm (F2). Moderately developed, cylindrical, and elongated. **Functions**. M30 is the retractor of the prothorax. The contraction of M30 pulls the prothorax closer to the mesothorax. Removing M30 does not result in the loss of clicking mechanism (see Table 2); however, it is not certain if M30 improves the clicking performance.

**M32x** (IIdvm8?): *M. dorso-ventralis*. **O:** mesothoracic axillary sclerites (?); **I:** lateral inflected area of the mesoventrite (Vt II). Slender and cylindrical. Not directly involved in the clicking mechanism.

**M33** (IItpm2): *M. noto-pleuralis*. **O:** first phragma (1Pm); **I:** pleural arm of the mesopleuron (PlA). Moderately developed, cylindrical, and short. **Functions**. M33 is usually the elytral levator; the contraction of M29 and M33 opens the elytra by turning the mesoscutellum forwards [45]. It is unknown if M33 is involved in the clicking mechanism.

**M36** (IItpm9): *M. pleura-alaris*. **O:** pleural arm of the mesopleuron (PlA); **I:** third axillary sclerite of the mesothorax (Ax3). Extremely weakly developed, cylindrical. Not involved in the clicking mechanism.

**M37** (IIspm2): *M. furca-pleuralis*. **O:** mesofurcal arm (F2); **I:** lower part of the pleural ridge of the mesopleuron (PlR). Weakly developed, fan-shaped. Not involved in the clicking mechanism.

**M40** (IIdvm4, 5): *M. noto-coxalis*. **O:** posterolateral part of the mesonotum (N II); **I:** posterior rim of the mesocoxa (Cx2). Moderately developed, cylindrical. M40 is attached to the clicking-related structure (mesonotum), but it is unknown if M40 is involved in the clicking mechanism.

**M41** (IIpcm4): *M. episterno-coxalis*. **O:** mesanepisternum (Es2); **I:** anterolateral rim of the mesocoxa (Cx2). Moderately developed, fan-shaped. Not involved in the clicking mechanism.

**M45** (IIscm4): *M. furca-coxalis lateralis*. **O:** mesofurcal arm (F2); **I:** posterolateral rim of the mesocoxa. Weakly developed, tapered ventrally. Not involved in the clicking mechanism.

**M46** (IIscm2): *M. mesofurca-coxalis posterior*. **O:** mesofurcal arm (F2); **I:** posterior rim of the mesocoxa (Cx2). Weakly developed, cylindrical. Not involved in the clicking mechanism.

**M48** (IIpcm6): *M. episterno-trochanteralis*. **O:** mesanepisternum (Es2); **I:** trochanteral tendon. Moderately developed, cylindrical. Not involved in the clicking mechanism.

(3) **Metathoracic Musculature** (not directly involved in the clicking mechanism; Figure 7, Figure 8, Figure 9 and Figure 10)

**M60** (IIIdlm1): *M. metanoti primus*. **O:** second phragma (2Pm) and the middle part of the metathoracic prescutum (Prs3); **I:** median lobe of the third phragma (3Pm-ML) and postnotum (Pn3). Strongly developed and cylindrical. 

**M61** (IIIdlm2): *M. metanoti secundus*. **O:** middle part of the metascutum (Sct3); **I:** lateral process of the third phragma (3Pm-LP). Strongly developed, oblique, and cylindrical. 

**M64** (IIIdvm1): *M. dorsoventralis primus*. **O:** median part and median ridge of the metaventrite; **I:** metathoracic prescutum (Prs3). Strongly developed, oblique, and cylindrical. 

**M66** (IIIdvm8) *M. dorsoventralis tertius*. **O:** lateral arm of the metafurca (LA); **I:** lateral process of the third phragma (3Pm-LP). Slender and tapered ventrally. 

**M67** (IIItpm2): *M. pleura-praealaris*. **O:** prealar sclerite (Pra); **I:** pleural ridge of the metapleuron (PlR). Small, conical. 

**M69** (IIItpm3): *M. noto-basalaris*. **O:** lateral part of the metathoracic prescutum (Prs3); **I:** basalar disc (BaD). Short, small, and cylindrical. 

**M71** (IIItpm7, 9): *M. pleura-alaris*. **O:** metanepisternum (Es3); **I:** a small sclerite in the membrane under the third axillary sclerite. Short, small, triangular, and divided into three branches on the metepimeron. 

**M73** (IIIspm1): *M. sterno-basalaris*. **O:** lateral part of the metaventrite (Vt III); **I:** basalar disc (BaD). Strongly developed, situated laterally to M64, and cylindrical.

**M74** (IIIdvm2): *M. noto-trochantinalis*. **O:** anterior part of the metascutum (Sct3); **I:** trochantinal disc. Strongly developed, situated posterior and parallel to M64, and cylindrical.

**M75** (IIIdvm4): *M. noto-coxalis anterior*. **O:** middle part of the metascutum (Sct3); **I:** inner surface of the metacoxa (Cx3). Strongly developed, situated posteriorly and parallel to M64, and cylindrical. 

**M76** (IIIdvm5): *M. noto-coxalis posterior*. **O:** lateral margin of the metascutum (Sct3); **I:** inner surface of the metacoxa (Cx3). Slender and cylindrical. The insertion of M76 in *C. auratus* is slightly different from those of other beetles studied, which is situated at the posterior metacoxal rim (based on [16]). 

**M78** (IIIpcm3): *M. coxa-basalaris*. **O:** anterior margin of the metacoxa (Cx3); **I:** basalar disc (BaD). Slender, cylindrical, and situated anterior and parallel to M76. 

**M79** (IIIdvm6): *M. coxa-subalaris*. **O:** inner surface of the metacoxa (Cx3); **I:** subalar disc (Sa). Strongly developed, cylindrical, and situated posterior and parallel to M78.

**M81** (IIIscm1): *M. furca-coxalis anterior*. **O:** stalk of the metafurca (Stk); **I:** anteromesal rim of the metacoxa (Cx3). Moderately developed, short, and tapering laterally.

**M82** (IIIscm4): *M. furca-coxalis lateralis*. **O:** ventral flange of the metafurca (VF); **I:** a process on the anterolateral rim of the metacoxa (Cx3). Moderately developed, transverse, long, and conical.

**M83a** (IIIscm2): *M. metafurca-coxalis posterior*. **O:** dorsal surface of the lateral arm of the metafurca (LA); **I:** posterior rim of the coxa (Cx3). Moderately developed, broad, and flattened.

**M83b** (IIIscm3): *M. metafurca-coxalis posterior*. **O:** ventral surface of the stalk of the metafurca (Stk); **I:** mesal part of the posterior rim of the metacoxa (Cx3). Weakly developed and conical.

**M85** (IIIscm6): *M. furca-trochanteralis*. **O:** lateral arm of the metafurca (LA); **I:** trochanteral tendon. Slender and cylindrical.

#### 3.1.7. Experiment 1. Testing Functions of Essential Clicking-Related Muscles and Sclerites

(1) **Clicking-Related Muscles**

We tested the functions of possible clicking-related muscles by cutting them from their attachments to the skeleton (see Table 2 and Section 2 ‘Materials and Methods’: Experiment 1). As a result, it appears that only M2 and M4 are critical muscles that enable the clicking mechanism: removing either of them resulted in the loss of the clicking ability. 

Removing M2 in *C. auratus* resulted in the loss of the ability to bend the prothorax dorsally (back-arching), and, therefore, the clicking mechanism was not functional. However, if the prothorax was pushed dorsad manually, the beetle could perform a single click. Removing M4 resulted in the individuals not clicking or clamping the thorax. The removal of various other muscles did not result in the loss of the clicking ability (see Table 2).

(2) **Clicking-Related Sclerites**

We tested the functions of the possible clicking-related sclerites by cutting them off the body (see Table 3 and Section 2 ‘Materials and Methods’: Experiment 1). It turned out that the following structures are essential for the clicking mechanism: the prosternal rest of the mesoventrite (PRM), the prosternal process (PP), the elytra, and the posterodorsal evagination of the pronotum (PdE). Removing any of them resulted in the loss of the normal clicking mechanism. The posterior angles (PA) of the pronotum are not essential for the clicking mechanism. The immobilization of the head also did not result in the loss of clicking mechanism.

**Table 2:** The influence of removing the thoracic muscles on the clicking mechanism in Elateridae.

The Muscle Removed	The Species Tested	The Ability to Click after the Operation
**M2a + M2b** (cut at posterior ends)	*C. auratus* (n = 1); *Actenicerus* sp. (n = 1); *Cryptalaus larvatus* (n = 1); *Cardiophorus* sp. (n = 1); *Melanotus* sp. (n = 4); and *Sternocampsus coriaceus* (n = 1).	Unable to click. Unable to bend the prothorax dorsad. If the prothorax was pushed dorsad manually, individuals could latch and click.
**M4** (cut at posterior ends)	*C. auratus* (n = 1); *Cryptalaus larvatus* (n = 1); *Melanotus* sp. (n = 2); and *Sternocampsus coriaceus* (n = 1).	Unable to click or interlock the thorax.
**M2b** (cut at anterior ends)	*C. auratus* (n = 1, c = 3); *Ludioschema obscuripes* (n = 1, c = 3).	Able to click.
**M1+ M2a + M8** (cut at anterior ends)	*C. auratus* (n = 1, c = 3); *Actenicerus maculipennis* (n = 1, c = 10); *Ludioschema obscuripes* (n = 2, h = 22.5 cm); *Melanotus* sp. (n = 1, h = 20.0 cm); and *Priopus* sp. (n = 1, h = 22.5 cm).	Able to click.
**M5+ M6+ M7** (cut at anterior ends)	*C. auratus* (n = 1, c = 3); *Ampedus* sp. (n = 1, c = 3); and *Melanotus* sp. (n = 1, h = 4.0 cm).	Able to click.
**M4x+ M11** (cut at posterior ends)	*C. auratus* (n = 1, h = 6.0 cm); *Melanotus* sp. (n = 3, h_1_ = 4.0 cm, h_2_ = 3.5 cm, and h_3_ = 15.5 cm); and *Pectocera fortunei* Candèze (n = 1, h = 2.0 cm).	Able to click.
**M30** (cut at middle)	*Cryptalaus larvatus* (n = 2, c = 3); *C. auratus* (n = 1, c = 3).	Able to click.

n = number of individuals tested; h = jumping heights after removing muscle, based on the average value of 5 jumps; and c = number of clicks observed. Jumping height data were not available in some individuals because they did not want to jump when injured. However, their clicks were observed when their abdomen was gently touched or squeezed by human hands.

**Table 3:** The influences of destructions of sclerites on the clicking mechanism in Elateridae.

The Structures Removed	The Species Tested	Influence on the Clicking Mechanism
**Prosternal rest of the mesoventrite (PRM)**	*C. auratus* (n = 1), *Campsosternus gemma* (n = 1), and *Silesis* sp. could not click; *Melanotus* sp. (n = 1, c = 3) and *Ludioschema obscuripes* (n = 1, c = 3) could click weakly.	Clicking mechanism was disrupted; the PP was stuck on the anterior cut edge of the mesoventrite.
**Prosternal process (PP)** (posterior part including the entire friction hold was removed)	*C. auratus* (n = 1), *Ludioschema obscuripes* (n = 1), and *Silesis* sp. (n = 1) could not click; *Melanotus* sp. (n = 1, c = 3) could click weakly.	Clicking mechanism was disrupted; the PP was stuck on the PRM.
**Elytron** (only one elytron was removed; auxiliary sclerites were not removed)	*C. auratus* (n = 1), *Pectocera fortunei*, *Ludioschema obscuripes* (n = 2), *Melanotus* sp., and *Silesis* sp. were all unable to click.	Clicking mechanism was disrupted; the PP was dislocated in the loading phase.
**Posterodorsal evagination of the pronotum (PdE)** (anterior bulged area was retained)	*C. auratus* (n = 2, c = 3), *Ludioschema obscuripes* (n = 1, c = 3), and *Melanotus* sp. (n = 1, c = 3) could click weakly; *Priopus angulatus* (n = 1, c = 3) could not click.	Clicking mechanism was disrupted; the loading motion was greatly weakened.
**Posterior angles of the pronotum (PA)**	*C. auratus* (n = 1, c = 3), *Ludioschema obscuripes* (n = 1, c = 5), and *Ludioschema dorsale* (n = 1, c = 3) could click normally.	Clicking mechanism was not affected.
**Head was immobilized using epoxy resin (in the retracted position)**	*C. auratus* (n = 2, c = 3) and *Ludioschema obscuripes* (n = 1, c = 3) could click normally.	Clicking mechanism was not affected.

n = number of individuals tested; c = number of clicks observed.

#### 3.1.8. The Promesothoracic Interlocking Mechanism

The promesothoracic interlocking mechanism is present in members of various families of the series Elateriformia, and it provides a morphological basis for the clicking mechanism in well-sclerotized elaterids [46]. 

The general shape of the posterior part of the prothorax is complementary to the mesothorax. When M4 contracts the mesothoracic sclerites can fit into the socket-shaped prothorax, so the promesothoracic gap can be interlocked precisely and tightly. In *C. auratus*, several pairs of thoracic structures can adapt to each other and form conformal contact. These complementary structures are identified in Table 4.

A defensive promesothoracic interlocking behavior was observed in this study: when we stretched a small brush into the promesothoracic gap of *C. auratus*, it immediately interlocked the thorax whenever the brush touched the structures. This behavior was also observed in *Sinelater perroti* (Tetralobinae), which represents one of the largest click beetles (body length 5.5–6.0 cm, width 1.7–1.9 cm, n = 5; live weight 6.38 g, n = 1). The fingers of two of our colleagues were accidentally injured (with minor bleeding) by the interlocking of the thorax. 

We also observed thoracic interlocking behaviors in the following representatives of Elateriformia: *Callirhipis* sp. (Dryopoidea, Callirhipidae, n = 2), *Eulichas* cf. *funebris* (Dryopoidea, Eulichadidae, n = 2), and *Chalcophora yunnana* (Buprestoidea, Buprestidae, n = 5). In *Callirhipis* sp. the prosternal process (PP) and mesoventral cavity (MsC) are extremely weakly developed; the posterodorsal evagination (PdE) and posteroventral evagination (PvE) are present and well-developed; the basal lobe of the elytron (BEL) is moderately sclerotized and produced anteriorly; and the basal elytral groove (BEG) is well-developed. In *Eulichas* cf. *funebris*, the PP, MsC, PdE, and PvE are present and weakly developed; the BEL is moderately sclerotized and produced anteriorly; and the BEG is well-developed. In *Chalcophora yunnana*, the PP, MsC, and PdE are present and well-developed; the PvE is absent; the BEL is strongly sclerotized; and the BEG is well-developed.

This tight and precise closure of the promesothoracic gap is also present in other beetles. It may be essential to protect against natural enemies, as the promesothoracic membrane is vulnerable. For example, according to Jordan et al. [47] the preferred site of the oviposition of *Microctonus brassicae* (Haeselbarth) (Hymenoptera: Braconidae) was the promesothoracic gap of the flea beetle *Psylliodes chrysocephala* L. (Coleoptera: Chrysomelidae). The well-sclerotized thoracic skeleton in many Elateriformia beetles may help them escape even larger natural enemies. This is because the powerful clamping of their pro- and mesothorax may cause injury to their predators.

### 3.2. The Jumping Mechanism of Campsosternus auratus

#### 3.2.1. The Jumping Process

The click beetle lies on the ground with an inverted position (ventral surface facing upward). The typical jumping process lasts 0.85–1.22 s, based on five jumps of five individuals. The process could be divided into the following four major phases: latching, loading, take-off, and airborne phases (adapted from Bolmin et al. [21,23]), and they are described as follows (Figure 11 and Figure 12, Supplementary Video S3):

(1) **Latching Phase**

The latching phase consists of the back-arching [13] and latching motions. The beetle lies on its dorsum on the ground; its pronotum rotates about the thoracic hinge dorsally, and the body reaches the back-arched position. This motion is enabled by the M2 muscle, as removing this muscle resulted in the loss of back-arching ability. The anterolateral region of the mesonotum (AR) and posterodorsal evaginations of the pronotum (PdE) constitute the thoracic hinge; the pronotum rotates around it to locate and set-up the latching position. At the end of the latching phase the friction hold (FH) of the prosternal process is set onto the prosternal rest of the mesoventrite (PRM), while the antennae and legs are retracted and placed close to the ventral side of the body.

The typical latching phase lasts 0.23–0.80 s, based on five jumps of five individuals. The angle between the pro- and mesothorax at the latched position is at 190–194° (based on two typical jumps of two individuals).

(2) **Loading/Contraction Phase**

As the friction hold (FH) of the prosternal process is temporarily locked on the prosternal rest of the mesoventrite (PRM), when M4 contracts, the thoracic structures (e.g., skeleton, elastic cuticles, and muscle tendons) are deformed. By these means, enormous tension is built up, and the elastic energy is stored in the deformed thoracic structures. In the loading phase the prealar bridge (PaBr) is significantly pulled posterad when M4x contracts. We hypothesize that the contraction of M4x helps to strengthen the thoracic hinge.

The following deformations could be observed in the loading phase (Figure 13E; see Section 3.2.5, ‘Experiment 2’, for more information): the prosternal process is pushed ventrally; the anterior part of the prosternum is levered dorsally. The anterior part of the mesonotum is pulled dorsally; and the posterior part of the mesoscutellum (Scl2) is slightly lowered and pressed onto the base of the elytra. These deformations were observed in both *C. auratus* and *Sinelater perroti*.

The typical loading phase lasts 0.15–0.72 s, based on five jumps of five individuals. The angle between the pro- and mesothorax in a fully loaded position is 198–200° (based on two typical jumps of two individuals).

(3) **Take-Off Phase**

At the beginning of the take-off phase the accumulation of tension and the deformation of thoracic structures make the latching system yield (i.e., triggering). The take-off phase starts with the prosternal friction hold (FH) disengaging from the prosternal rest of the mesoventrite (PRM); the previously stored, enormous elastic energy is translated into kinetic energy. The prosternal process slides into the mesoventral cavity abruptly; in the meantime, the anterior and posterior parts of the body rotate about the thoracic pivot, bending ventrad rapidly (i.e., a jack-knifing movement [13]). 

The ventrad bending movement has a great velocity, which results in a quick elevation of the centers of gravity of the anterior and posterior parts of the body [13]. Therefore, according to the law of the conservation of momentum, the middle part of the body tends to move in the opposite direction (i.e., moving dorsally) rapidly. However, this results in the base of the elytra being pushed onto the ground. Eventually, the counterforce from the ground acts on the base of the elytra and pushes up the beetle in a few milliseconds. 

The typical take-off phase occurs in 0.0016–0.0026 s (based on five jumps of five individuals), ending with the body leaving the ground.

(4) **Airborne Phase**

After the beetle leaves the ground it rotates (see the following ‘somersault’ section). At the beginning of this phase the anterior and posterior parts of the body bend ventrally at high speed (i.e., jack-knifing movement), and subsequently they swing back and forth in an oscillating mode [13,23]. This rapid oscillation motion was recently studied in-depth by Bolmin et al. [23]. They described the oscillation as the recoil of elastic structures and the nonlinear damping process, which dissipates the kinetic energy and gradually slows down the body’s anterior and posterior parts.

Three to four cycles of oscillation were observed based on ten typical jumps. Three other click-beetle species were also tested for comparison purposes: six to seven cycles of oscillation based on four typical jumps in *Cryptalaus larvatus* (Candèze) (Agrypninae); six to seven cycles based on three typical jumps in *Ludioschema obscuripes* (Elaterinae); and five cycles based on one typical jump in *Sinelater perroti* (Tetralobinae). During the oscillation of the prothorax, the prosternal process slides in and out of the mesoventral cavity repeatedly; in the meantime, the prothorax swings ventrad and dorsad alternatively around the thoracic hinge (Figure 13F). 

The oscillation lasts 0.006–0.008 s, with a frequency ranging from 200 to 270 HZ (based on five typical jumps of five individuals). The minimal angle between the pro- and mesothorax occurs in the first oscillation cycle, which is 159–161° in *C. auratus* (based on two typical jumps of two individuals), 148° in *Sinelater perroti* (based on one jump), and 120° in *Cryptalaus larvatus* (based on one jump). When the oscillation finishes, the pro- and mesothorax angle is 167–170°. The promesothoracic gap gradually closes during the oscillation. The promesothoracic gap is entirely closed in 0.12–0.20 s right after the end of the oscillation (observed in five typical jumps of five individuals). At the end of a regular airborne phase, the promesothoracic gap opens, and the legs stretch out before hitting the ground.

The typical airborne phase lasts 0.20–0.32 s based on five jumps of five individuals. 

#### 3.2.2. The Jumping Performance

**Righting Behavior**

The righting behavior of five individuals was observed. The probability of righting themselves was recorded as follows: individual one: 66% (based on 50 jumps); individual two: 50% (based on ten jumps); individual three: 50% (based on ten jumps); individual four: 90% (based on ten jumps); and individual five: 60% (based on ten jumps). The data seem irregular, but all the tested individuals had a probability higher than or equal to 50%.

**Jumping Height**

The jumping height ranges from 5.4 cm to 14.7 cm, based on the five individuals tested. The jumping heights for each individual are listed as follows: individual one: 14.7 cm (average of 20 jumps); individual two: 5.4 cm (average of 10 jumps); individual three: 6.9 cm (average of 10 jumps); individual four: 7.2 cm (average of 10 jumps); and individual five: 9.8 cm (average of 10 jumps).

A test of one individual showed that it was able to jump more than 70 times within 10 min without prominent fatigue. The average height of the first ten jumps was 14.8 cm; the average height of the last ten jumps was 14.6 cm.

**The Somersaults during the Airborne Phase**

In total, seven individuals were tested, and five jumps of each individual were recorded by high-speed filming (Figure 11B).

(1) Roll (rotate about the longitudinal axis). Rolling was present in six individuals and absent in one. In typical jumps, the body turns 360–980°. The rolling orientation is constant in the same individual. We hypothesize that the elytra shape could affect the rolling direction, as the counterforce from the ground acts on the base of the elytra.
(2) Pitch (rotate about the transverse axis). All jumps of the seven individuals rotated head over tail. The body turns 360–540° in typical jumps.
(3) Yaw (rotate about the dorsoventral axis). Yaw was absent in all seven individuals. According to Evans [13], yaw was also lacking in the jumps of *Athous haemorrhoidalis*.

**Escaping Behavior**

We encountered and observed the escaping behavior of an individual of *C. auratus* in the wild; it dropped to the litter on the ground when we approached it. When it was captured and seized by fingers, it clicked repeatedly and tried to escape with the help of the vibration and the impact it caused. We found that the body of *C. auratus* is quite hard to grasp when it is clicking consecutively. Based on our previous field experience, it is also not easy to capture other click beetles while they are clicking, as many of them have slippery body surfaces and ovoid shapes. 

#### 3.2.3. What Triggers the Jump?

Evans [13] proposed several possible explanations for the triggering of the clicks: (1) the protraction of the head; (2) the contraction of M2; (3) the contraction of M30; and (4) the building-up of tension. We suggested that the building-up of elastic energy and the deformation of thoracic structures make the prosternal process slip from the prosternal rest of the mesoventrite (PRM) and trigger the clicks. This is supported by the following observations:

(1) Protraction of the head does not always occur in the jumps of *C. auratus*; protraction of the head was absent in *Sinelater perroti* (four jumps of one individual were observed); (2) we tested two individuals of *C. auratus* by immobilizing their head using epoxy resin when their heads were retracted; they could still click; and (3) a specialized trigger muscle was not found in *C. auratus*. Based on ‘Experiment 1’ in this study, although removing M2 (M2a + M2b) disabled the back-arching ability of the beetle, it still could latch and click if one pushed the beetle’s prothorax dorsally; the tests on several other possible clicking-related muscles also showed that none of them were the specialized trigger muscle.

#### 3.2.4. What Slows down the Oscillation of the Body?

At the beginning of the airborne phase, the anterior and posterior portions of the body swing back and forth in an oscillating mode [13,23]. This oscillation motion was recently studied in detail by Bolmin et al. [23]. 

Based on our observations, several resistances may contribute to slowing down the oscillation. (1) The oscillation happens right after the loading phase, and the oscillation duration is extremely short (0.006–0.008 s). The promesothoracic gap gradually closes during the oscillation. We assume that M4 is still in a contracted state. The contracted M4 tends to bend the prothorax ventrad and close the thoracic gap. Therefore, a part of the kinetic energy may be absorbed by M4 each time the prothorax swings dorsad during the oscillation. (2) The repeated deformation and recoil of thoracic structures may dissipate part of the kinetic energy. (3) The friction between the conformal structures (see Table 4) may dissipate part of the kinetic energy. The prothoracic posterodorsal evaginations (PdE) are pressed on the anterolateral region of the mesonotum (AR) and rotate back and forth around it, repeatedly. The prosternal process (PP) repeatedly slides in and out of the mesoventral cavity (MsC). Under these resistances the kinetic energy gradually turns into thermal energy and dissipates into the thoracic sclerites and contents.

Evans [13] proposed that, after the take-off, the clicking process might be stopped by the ‘bumper’ (i.e., the profurcal base (FB)) colliding with the ‘buffer’ (i.e., the prosternal rest of the mesoventrite (PRM)). However, this theory raises several doubts: (1) It is uncertain if the ‘bumper’ could reach the ‘buffer’ in the clicking process. Based on dissections, the FB can only come into contact with the PRM when the pro- and mesothorax are tightly interlocked together; and in the clicking process, there is still a gap between the pro- and mesothorax (approximately 0.8–1.0 mm in length, measured in two specimens in the high-speed filming, which approximately equals 2/3 of the diameter of the coxa in lateral view). (2) When the prothorax is abruptly bent ventrad towards the mesothorax, the angle between them is 159–161° in *C. auratus* (based on two typical clicks of two individuals), 148° in *Sinelater perroti* (based on one click of one individual), and 120° in *Cryptalaus larvatus* (based on one click). It is uncertain if the FB could reach the PRM when the pro- and mesothorax are at such an angle. (3) There are multiple oscillations between the pro- and mesothorax, and the relative position between the FB and PRM changes at each oscillation.

#### 3.2.5. Experiment 2. Observation of the Deformation of Thoracic Structures and Elastic Energy Storage in the Loading Phase

In the loading phase the skeletal structures are deformed when M4 contracts (Figure 13). ‘Experiment 2’ (see Section 2 Materials and Methods) was conducted to reveal the visible deformations. The deformation of the prosternum and mesonotum is most significant; they both play the role of a biological spring, which deforms in the loading phase to store elastic energy and recoils in the take-off phase to release said energy. Other sclerites have minimal deformations, which are hard to observe, such as the pronotum, mesopleuron, mesoventrite, and base of the elytra. Evans [13] also pointed out that the elastic energy could be stored in the temporary distortion of elastic cuticles between sclerites and the elastic component of muscles (such as apodeme and tendons).

The visible deformation of the thorax of *C. auratus* is described as follows:

1. Deformation of the prosternum (Figure 12B and Figure 13E). The high-speed filming of the lateral view of two jumps of two individuals was analyzed. The distance between the dorsal apex of the prosternal process (PP) and the apex of the posterior angle of the pronotum (PA) in the lateral view is denoted as *PP-PA* (indicated in Figure 12B). Before the loading phase, *PP-PA* is at its minimum: 3.81 mm (individual one), 3.97 mm (individual two). At the end of the loading phase the PP is pushed ventrad, and *PP-PA* is at its maximum: 4.65 mm (individual one), 4.60 mm (individual two). The change in the *PP-PA* during the loading phase is 0.63–0.84 mm.

Evans [13] found that, during the loading phase, the pronotosternal suture was pushed open slightly, particularly at the anterior part. This phenomenon was also present in *C. auratus*. Both the bent prosternum and stretched elastic cuticle of the pronotosternal suture contribute to the storage of elastic energy. On the other hand, the same phenomenon is absent in *Sinelater perroti*, in which the prosternum and hypomeron are entirely fused, and the pronotosternal suture is strongly sclerotized.

2. Deformation of the mesonotum (Figure 13E). In the loading phase the anterior part of the mesonotum is deformed and pulled dorsad towards the pronotum by the contraction of M4 (Figure 13E). The mesoscutellar shield (SclS2) inclines backward and is pushed onto the base of the elytra, and it restores the original position when the loading motion is ended. The displacement of the mesoscutellar shield also confirms the deformation of the anterior part of the mesonotum. We hypothesize that the sophisticated saddle-like shape of the mesonotum may have an advantage for the storage and swift release of elastic energy. These deformations were observed in both *C. auratus* and *Sinelater perroti*.

An additional test was conducted: We removed the elytra of an individual of *C. auratus*. Without the elytral mesoscutellar interlocking, the constraining at the posterior part of the mesonotum was absent. In the loading phase, the position of the mesonotum was much more dramatically changed by M4; the anterior part was much more significantly raised dorsad; and the rear part was greatly levered ventrad.

3. Deformation of the pro- and mesothoracic intersegmental contents. When M4 contracts, the intersegmental membrane (Figure 1F,G: Meb) between the pro- and mesothorax and the internal thoracic contents shift dorsad. This phenomenon was named the ‘soft cuticle contraction’ in Bolmin et al. [23]. The high-speed film of the lateral view of two jumps of two individuals was analyzed. The distance between the Meb and ventral surface of the prosternal process (PP) in lateral view was measured; it is denoted as *Meb-PP* (indicated in Figure 12B). Before the loading phase the *Meb-PP* is at its minimum: 0.18–0.31 mm. At the end of the loading phase the Meb moves dorsad, and the *Meb-PP* is at its maximum: 0.96–1.03 mm. The displacement of the Meb in the dorsoventral axis is 0.65–0.85 mm. The displacement of the Meb in the longitudinal axis is 0.81–0.84 mm.

#### 3.2.6. Recording of the Clicking Sounds

Evans [13] suggested two possible ways to explain the origin of the audible clicking sounds generated by click beetles: the sudden slipping of the peg over its friction hold or the violent impact of the prothoracic bumper on the ‘mesoventral buffer’. Here, we propose that the oscillation of the thorax might be the source of the audible click.

The clicking sounds of one individual of *C. auratus* were recorded. The oscillograms of the clicking sound show that the audible sound lasts 0.007–0.015 s (based on three clicks) (see Section 2 ‘Materials and Methods’ for the experimental method). This is very close to the oscillation time of the body (0.006–0.008 s) calculated based on high-speed filming. The recording shows that the frequency of the clicking sound ranges from 160 HZ to 250 HZ (based on three clicks). The oscillation frequency of the body was also calculated based on high-speed filming (three clicks of three individuals were filmed). This showed that the oscillation frequency ranges from 200–270 HZ. The frequency of the clicking sound and the oscillation movement are also quite close to each other.

As sound is the acoustic wave produced by the vibration of objects, we noted that the oscillation of the thorax was a probable source of the audible clicking sounds. However, an additional future experiment is needed to confirm this hypothesis, such as recording the clicking sound synchronous with the high-speed filming.

**Figure 14:**
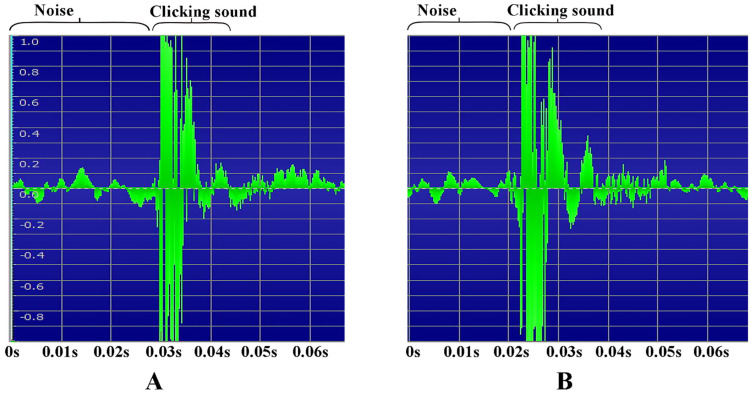
The oscillograms of the clicking sound of *Campsosternus auratus*. (**A**,**B**) show two clicking sounds of an individual; the oscillogram photograph was generated using GOLDWAVE software. (**A**) The clicking sounds last approximately 0.007 s; the frequency is approximately 250 HZ. (**B**) The clicking sound lasts approximately 0.015 s; the frequency is approximately 200 HZ.

## 4. Discussion

With the aid of 3D reconstruction and high-speed filming techniques, this study described and presented the thoracic skeleton and musculature of *C. auratus*, as well as the functions of these structures in the jumping and promesothoracic interlocking mechanisms. The thorax of *C. auratus* is characterized by the prominently elongated prosternal process, evaginated posterior part of the pronotum, saddle-shaped mesonotum, deeply concaved mesoventrite, and specialized elytral base. These structures are all highly specialized, strongly sclerotized, and well-adapted to each other. The larger and longer pronotum provides the space for the enormous M4 muscle, and the longer prothorax and ‘hind body’ ensure their respective centers of gravity can be raised as much as possible [17]. The specialized and robust thoracic structures enable the clicking mechanism, the absorption of the redundant kinetic energy, and the protective promesothoracic interlocking behavior. The identification of the skeleton and musculature is the basis for understanding the jumping mechanism correctly. What is more, the three-dimensional data contain even more information to reveal the function of each structure.

We hypothesize that the sophisticated arched shape of the mesonotum has an advantage for elastic energy storage and release in the clicking mechanism. Reports of similar biological springs can be found in previous studies. For example, locusts have a semilunar process on the distal part of the hind femur for elastic energy storage and release [2]. The raptorial appendages of mantis shrimps also utilize a saddle-shaped structure as an exoskeletal spring [48,49]. Tadayon et al. [50] unveiled that the saddle of mantis shrimps (stomatopods) stores a high density of elastic energy and prevents stress concentration during loading. The morphology of the mesonotum of click beetles is quite close to the saddle-shaped structure of stomatopods. Future studies are required to further understand the mechanics of click beetles’ mesonotum.

The deformation of the pro- and mesothoracic intersegmental membranes and internal contents was previously described as ‘soft cuticle contraction’ and ‘soft cuticle displacement’ in Bolmin et al. [23]. They also revealed that the ‘soft cuticle’ area contributes to the spring mechanism through rapid recoil. Here, we propose a more accurate morphological description of the phenomenon: the actual primary structures that contribute to the spring mechanism are the M4 and saddle-shaped mesonotum on the dorsal side of the ‘soft cuticle’; the deformation and displacement of the intersegmental membranes and contents is caused by the contraction of M4 (and probably M30), the raising of the anterior part of mesonotum, and the change in hemolymph pressure. All these deformations of structures can be restored when the beetle aborts the loading motion.

One of the unresolved questions regarding the clicking mechanism is how the brain and nerve system sustain the impact caused by the clicking. Similar mechanisms were investigated in detail in woodpeckers, as their head and neck also sustain repeated blows during the pecking of wood. Their anti-impact ability is attributed to the enlarged brain volume and specialized skull structure, and the interesting anti-impact mechanisms of woodpeckers have attracted wide attention in the fields of ornithology, medicine, and materials science [51,52,53]. Although the internal structure of click beetles is very different and much smaller, its nerve system also sustains repeated substantial impact in a short period of time. According to Evans [17], the acceleration inflicted upon the brain of *Athous haemorrhoidalis* peaks at 2300 g (i.e., approximately 23,000 ms^−2^). This is an exciting aspect, which requires future investigation.

Another interesting question is whether all clicking beetle groups share the same clicking mechanism or if they differ in some aspects. Within Elateroidea, the groups with a well-developed clicking mechanism, such as Elateridae, Eucnemidae, Throscidae, and Cerophytidae (i.e., Elateroidea *sensu stricto*, or ‘clicking elateroids’), were originally thought to form a monophyletic lineage (e.g., [39]); however, recent molecular phylogenetic studies show that Elateridae are more related to the soft-bodied families (i.e., some former Cantharoidea) than to the remaining clicking groups (e.g., [28,54]). The in-depth investigation of the clicking mechanism in Eucnemidae, Throscidae, and Cerophytidae was beyond the scope of our study. Instead, we focused on Elateridae. Our study shows that the general morphology of *C. auratus* is similar to that of *Elater sanguineus* and *Selatosomus aeneus* investigated by Larsén [16] and the *Athous haemorrhoidalis* investigated by Evans [13]. The jumping mechanism of *C. auratus* is similar to that of *Athous haemorrhoidalis* and several other species studied by Bolmin et al. [21,22,23]. One difference between the different species of Elateridae that we found was the function of the pronotosternal suture. Based on our study, the pronotosternal suture in *C. auratus* consists of an elastic cuticle; in the loading phase the elastic cuticle is stretched, and the anterior part of the prosternum is raised dorsally into the prothorax. On the other hand, in *Sinelater perroti,* the pronotosternal suture (PsS) is sclerotized, and the prosternum and hypomeron are entirely fused at their boundary. Therefore, the anterior part of the prosternum of *Sinelater perroti* is unable to deform or store elastic energy, as in some other species. Apart from the pronotosternal suture, the clicking mechanism of the different investigated click beetles seems to be similar. Further investigation of different species from yet-unstudied subfamilies and especially from morphologically specialized groups should be carried out, in order to provide insights into the comparative morphology of clicking-related structures within Elateridae.

## 5. Conclusions

In this study, we investigated the thoracic morphology of *C. auratus* and several hitherto unknown or less-studied aspects of the specialized clicking and jumping mechanisms, and we came to several interesting conclusions: (1) In a typical jump, *C. auratus* depends on the enormous M4 muscle to generate a colossal energy, and the deformation of specialized thoracic structures (e.g., the prosternum and mesonotum) to store it; the building-up of elastic energy and deformation of the thoracic structures trigger the explosive release of energy and raise the body in a few milliseconds. (2) We show that the destruction of several structures results in the loss of jumping ability; such as M2, M4, the posterior part of the prosternal process (PP), the prosternal rest of the mesoventrite (PRM), the base of the elytra (BEL), and the posterodorsal evagination of the pronotum (PdE). (3) The mesonotum is found to be critical for jumping: its complex saddle-like shape is specialized for the storage and abrupt release of elastic energy; it is also the thoracic hinge and rotation center of the clicking system. (4) A more accurate morphological description is provided for the ‘soft cuticle contraction’ phenomenon described in Bolmin et al. [23]. It is attributed to the contraction of M4 and deformation of the mesonotum and other thoracic structures. (5) We identified the probable resistances that slow down the ‘oscillation motion’ of the body. Additionally, our analysis does not support the theory proposed by Evans [13], that the profurcal base (FB) collides with the prosternal rest of the mesoventrite (PRM) to slow down the motion.

Although our study shows that the morphology and jump of *C. auratus* are similar to that of other click beetles, as reported in several previous studies [13,17,21,22], *Sinelater perroti* has a very different pronotosternal suture (PsS), which may result in an entirely different strategy of elastic energy storage and release. This shows that future in-depth investigation of other species may further help our understanding of the jumping mechanism and morphology in Elateridae.

## Figures and Tables

**Table 1:** Specimen information.

	Species Name and Numbers of Individuals Used	Subfamily/Family
**Dissected Elateridae specimens**	*Campsosternus auratus* (ethanol-preserved specimens, n = 11; dry specimens, n = 14)	Dendrometrinae
	*Sinelater perroti* (dry specimens, n = 2)	Tetralobinae
	*Phorocardius unguicularis* (Fleutiaux) (dry specimen, n = 1)	Cardiophorinae
**Specimens used for micro-CT scans**	*Campsosternus auratus* (n = 2)	Dendrometrinae
**Specimens used for high-speed filming and observation of jumping performance**	*Campsosternus auratus* (n = 24)	Dendrometrinae
	*Sinelater perroti* (n = 11)	Tetralobinae
**High-speed filming of Elaterid species used for comparison purposes**	*Agrypnus bipapulatus* (Candèze) (n = 2), *Agrypnus costicollis* (Candèze) (n = 1), *Cryptalaus berus* (Candèze) (n = 1), and *Cryptalaus larvatus* (Candèze) (n = 2)	Agrypninae
	*Cardiophorus* sp. (n = 1)	Cardiophorinae
	*Ludioschema obscuripes* (Gyllenhal) (n = 1), *Melanotus* sp. (n = 2)	Elaterinae
**Specimens used for ‘Experiment 1’ and ‘Experiment 2’**	*Campsosternus auratus* (n = 24), *Campsosternus gemma* Candèze (n = 1), *Actenicerus maculipennis* (Schwarz) (n = 2), *Pectocera fortunei* Candèze (n = 2), and *Sternocampsus coriaceus* Liu *et* Jiang (n = 2)	Dendrometrinae
	*Ampedus* sp. (n = 1), *Ludioschema dorsale* (Candèze) (n = 1), *Ludioschema obscuripes* (n = 5), *Melanotus* sp. (n = 18), *Priopus angulatus* (Candèze) (n = 1), *Priopus* sp. (n = 1), and *Silesis* sp. (n = 3)	Elaterinae
	*Cardiophorus* sp. (n = 1)	Cardiophorinae
	*Cryptalaus larvatus* (n = 5)	Agrypninae
	*Sinelater perroti* (n = 2)	Tetralobinae
**Specimens used for** **the recording of the clicking sounds**	*Campsosternus auratus* (n = 3)	Dendrometrinae
**Specimens used for** **an additional test to observe the displacement of the mesonotum in the loading phase**	*Campsosternus auratus* (n = 1)	Dendrometrinae
	*Sinelater perroti* (n = 1)	Tetralobinae
**Specimens used for observation of the interlocking mechanism of the thorax**	*Campsosternus auratus* (n = 4)	Agrypninae
	*Sinelater perroti* (n = 1)	Tetralobinae
	*Callirhipis* sp. (n = 2)	Callirhipidae
	*Eulichas* cf. *funebris* (Westwood) (n = 2)	Eulichadidae
	*Chalcophora yunnana* Fairmaire (n = 5)	Buprestidae

n = number of individuals used in this study.

**Table 4:** The complementary structures of the pro- and mesothorax of *Campsosternus auratus*.

Complementary Structures	Interaction of the Structures	Contributions to the Following Mechanisms
Flange of the elytral base (EBF) + posterodorsal groove of the pronotum (PGr)	Conformal contact	Thoracic interlocking.
Posteroventral evagination of the pronotum (PdE) + basal elytral groove (BEG)	Conformal contact	Thoracic interlocking.
Posteroventral evagination of the pronotum (PdE) + anterolateral region of the mesonotum (AR)	Conformal contact	Clicking and thoracic interlocking. The PdE and AR form the thoracic hinge for the clicking mechanism.
Posteroventral evagination of the pronotum (PvE) + mesanepisternum (Es2)	Conformal contact	Thoracic interlocking. Its involvement in the clicking mechanism is unknown.
Mesal basal part of the elytra + posterior part of the mesonotum	Conformal contact; interlock with each other	Clicking, thoracic interlocking, and elytral-mesoscutellar interlocking.
Friction hold (FH) of the prosternal process + prosternal rest of the mesoventrite (PRM)	Conformal contact	Clicking.
Prosternal process (PP) + mesoventral cavity (MsC)	Conformal contact	Clicking.

## Data Availability

Not applicable.

## Supplementary Materials

The following supporting information can be downloaded at: https://www.mdpi.com/article/10.3390/insects13030248/s1. Supplementary File S1: Specimen information. Supplementary File S2: Simplified 3D models of the exoskeleton and thoracic muscles of *Campsosternus auratus*. The following structures are shown on different pages: (1) exoskeleton and thoracic muscles, (2) thoracic exoskeleton, (3) meso- and metathoracic exoskeleton, (4) meso- and metathoracic exoskeleton (elytra removed), (5) exoskeleton of prothorax, and (6) mesonotum. Supplementary Video S3: High-speed filming of the jump of *Campsosternus auratus*. Video S3 consists of three videos. Video one: a general view of the trajectory of the jump (frame interval of 230 μs; exposure of 50 μs; 4347.8 fps; 2573 frames recorded; and a time span of 0.59179 s). Video two: close-up view of the body in the take-off phase (frame interval of 230 μs; exposure of 25 μs; 4347.8 fps; 47 frames recorded; and a time span of 0.01081 s). Video three: close-up view of the thorax in the loading and take-off phases (frame interval of 230 μs; exposure of 25 μs; 4347.8 fps; 2149 frames recorded; and a time span of 0.49427 s).
