# Supplementary material for: Functional Morphology of the Thorax of the Click Beetle Campsosternus auratus (Coleoptera, Elateridae), with an Emphasis on Its Jumping Mechanism
Source: Insects. 2022 Feb 28;13(3):248. doi: 10.3390/insects13030248 (PMC8955093; doi:10.3390/insects13030248)
Supplement: Supplementary file 1 [file insects-13-00248-s001.zip › Supplementary file S1. Specimen information.pdf]

Supplementary file S1. Specimen information

The Elateridae specimens used in the high-speed filming and the experiments are listed as follows. All specimens are housed in the Plant protection research center, Shenzhen polytechnic (SZPT).

(1) Dissected Elateridae specimens:

Ethanol preserved specimens of *Campsosternus auratus* (Drury): 2 individuals, China, **Guangxi**, Guilin, Zi-yuan County, 2021.VI.27, leg. Wenyu Ma. • 2 individuals, China, **Guangxi**, Hezhou, Mt. Er-zeng-shan, 2021.VI.20, leg. Yonglu Zhou. • 3 individuals, China, **Fujian**, Fuan, Shuyang Village, 368 m, 27°09′32″N,119°40′34″E, 2021.VI.20, leg. Xiuzhao Lin. • 1 individual, China, **Fujian**, Nanping, 2021.VI.4, leg. Hanpeng Lin. • 1 ethanol preserved specimen, China, **Hongkong**, Pat Sin Leng Country Park, Hok Tau, 2017.VI.11, leg. Jianzhao Wu. • 1 individual, China, **Guangdong**, Zhanjiang, 2021.V.5, leg. Yu Li. • 1 individual, China, **Hongkong**, Baxianling Park, Hesou, vi-11-2017, sweeping, leg. Jianzhao Wu.

Dry specimens of *Campsosternus auratus* (Drury): • 1 individual, China, **Guangdong**, Shenzhen, Mt. Wu-tong-shan, 2008.V.24, leg. Ziye Meng & Minbao Jiang. • 1 individual, China, **Guangdong**, Shaoguan, Nan-ling National Nature Reserve, 2011.VII-VIII, leg. Beishen Chen. • 5 individuals, China, **Guangdong**, Shenzhen, Mt. Dananshan, 2018.V.26, leg. Shengli Zhou. • 2 individuals, China, **Yunnan**, Honghe, Mt. Daweishan, 2016.VI.1, leg. Tianlong He. • 1 individual, China, **Guangdong**, Shenzhen, Da-nan-shan Park, 2018.V.26, leg. Ya Shi, dry specimen; immersed in 10% NaOH sulution for 24 hours before dissection • 2 individuals, China, **Guangdong**, Shenzhen, Da-nan-shan Park, 2018.V.26, leg. Ya Shi; immersed in 10% NaOH sulution for 24 hours before dissection. • 2 individuals, China, **Hongkong**, Heshou, Baxianling country Park, 2017.VI.27, sweeping, leg. Jianzhao Wu.

Dry specimens of *Sinelater perroti* (Fleutiaux): 2 individuals, China, **Guangdong**, Shaoguan, Nan-ling National Nature Reserve, 2011.VII-VIII, leg. Beishen Chen.

Dry specimen of *Phorocardius unguicularis* (Fleutiaux): 1 individual, China, **Yunnan**, Yingjiang, Sudian, Lao-ma-he, 2018.VI-VII, 2300m, leg. Weizong Liang, light trap.

(2) Specimens used for micro-CT scans:

Campsosternus auratus (Drury) with resting position: 1 female, ethanol preserved specimens, China, **Fujian**, Fuan, 2017.IV, leg. Yongying Ruan.

Campsosternus auratus (Drury) with back-arched position: 1 female, ethanol preserved specimens, China, **Guangxi**, Hezhou, Mt. Er-zeng-shan, 2021.VI.20, leg. Yonglu Zhou.

(3) Specimens used for high-speed filming and observation of jumping performance (some individuals were also used in Experiment 2)

In total, 121Gb files of high-speed filmings were recorded for *Camsosternus auratus*, 113Gb were recorded for other Elteridae species for comparison purposes.

Campsosternus auratus (Drury): 6 individuals, China, **Guangxi**, Hezhou, Mt. Er-zeng-shan, 2021.VII.1, leg. Yonglu Zhou. • 1 individual, China, **Guangxi**, Guilin, Zi-yuan County, 2021.VII.7, leg. Wenyu Ma. • 1 individual, China, **Hunan**, Huaihua, Chen-xi County, 2021.VII.2, leg. Gang Huang. • 4 individuals, China, **Hunan**, Huaihua, Chen-xi County, 2021.VII.10, leg. Gang Huang. • 1 individual, China, **Hunan**, Huaihua, Chen-xi County, 2021.VII.18, leg. Gang Huang (also used in Experiment 2). • 1 individual, China, **Fujian**, Fuan, Shuyang Village, 368 m, 27°09′32″N,119°40′34″E, 2020. VII.28, leg. Yongying Ruan• 2 individuals, China, **Fujian**, Fuan, Shuyang Village, 368 m, 27°09′32″N,119°40′34″E, 2021.

VII.15, leg. Xiuzhao Lin (also used in Experiment 2). • 4 individuals, China, **Fujian**, Nanping, 2021.VII.14, leg. Mengna Zhang (also used in Experiment 2). • 1 individual, China, **Zhejiang**, Hangzhou, Lin-an, 2021.VII.2, leg. Mengna Zhang. • 3 individuals, China, **Fujian**, Fuan, Shuyang Village, 368 m, 27°09′32″N,119°40′34″E, 2021. VII.5, leg. Xiuzhao Lin (also used in Experiment 2).

Sinelater perroti (Fleutiaux): 1 individual, China, **Fujian**, Nanping, Shao-wu, 2021.VII.15, leg. Chen Chen. • 2 individuals, China, **Guangxi**, Guilin, Zi-yuan County, 2021.VII.15, leg. Wenyu Ma, light trap. • 1 individual, China, **Guangdong**, Shao-guan, Che-ba-ling National Nature Reserve, Chebaling Village, 2021. VI.2, 24.703638°N, 114.151580°E, 487m, leg. Yongying Ruan, light trap. • 2 individuals, China, **Fujian**, Nanping, Mt. Mangdangshan, 850m, 2021.VI.3, leg. Yingchun Liu, light trap. • 4 individuals, China, **Fujian**, Nanping, Mt. Mangdangshan, 850m, 2021.VII.8, leg. Yingchun Liu, light trap. • 1 individual, China, **Guangdong**, Shao-guan, Che-ba-ling National Nature Reserve, 2020.VIII.23., Yongying Ruan, light trap.

The high-speed filmings of the following Elaterid species were also recorded for comparison purposes:

Agrypnus bipapulatus (Candèze): 2 individuals, China, **Guangdong**, Shaoguan, Che-ba-ling National Nature Reserve, administration center, 2020.VIII.23, leg. Yongying Ruan.

Agrypnus costicollis (Candèze): 1 individual, China, **Guangdong**, Shaoguan, Che-ba-ling National Nature Reserve, administration center, 2020.VIII.23, leg. Yongying Ruan.

Cardiophorus sp.: 1 individual, China, **Guangdong**, Shaoguan, Che-ba-ling National Nature Reserve, administration center, 2020.VIII.23, leg. Yongying Ruan.

Cryptalaus berus (Candèze): 1 individual, China, **Fujian**, Fuan, Shuyang Village, 368 m, 27°09′32″N,119°40′34″E, 2020.VII.29–31, leg. Yongying Ruan.

Cryptalaus larvatus (Candèze): 1 individual, China, **Guangdong**, Shaoguan, Che-ba-ling National Nature Reserve, administration center, 2020.VIII.23, leg. Yongying Ruan. • 1 individual, China, **Fujian**, Fuan, Shuyang Village, 368 m, 27°09′32″N,119°40′34″E, 2020.VII.29–31, leg. Yongying Ruan.

Ludioschema obscuripes (Gyllenhal): 1 individual, China, **Guangdong**, Shao-guan, Nan-ling National Nature Reserve, Babaoshan Station, 2020.VIII.28, 24.9323°N, 113.0191°E, 1000m, leg. Yongying Ruan, light trap.

Melanotus sp.: 1 individual, China, **Hunan**, Mang-shan National Nature Reserve, Cha-wang-gu, 2020.VIII.30, leg. Yongying Ruan. • 1 individual, China, **Fujian**, Fuan, Shuyang Village, 368 m, 27°09′32″N,119°40′34″E, 2020.VII.29–31, leg. Yongying Ruan.

(4) Specimens used for Experiment 1 and Experiment 2:

Campsosternus auratus (Drury): • 1 specimen, China, **Hunan**, Huaihua, Chen-xi County, 2021.VII.18, leg. Gang Huang. • 1 individual, China, **Hunan**, Huaihua, Chen-xi County, 2021.VII.18, leg. Gang Huang. • 2 individuals, China, **Fujian**, Fuan, Shuyang Village, 368 m, 27°09′32″N,119°40′34″E, 2021. VII.15, leg. Xiuzhao Lin； • 4 individuals, China, **Fujian**, Nanping, 2021.VII.14, leg. Mengna Zhang； • 3 individuals, China, **Fujian**, Fuan, Shuyang Village, 368 m, 27°09′32″N,119°40′34″E, 2021. VII.5, leg. Xiuzhao Lin. • 2 individuals, China, **Guangdong**, Qingyuan, Shi-men-tai National Nature Reserve, Qian-jin Station, 2021.V.26, 24.488679°N, 113.114489°E, 524m, leg. Yongying Ruan. • 6 individuals, China, **Guangdong**, Qingyuan, Shi-men-tai National Nature Reserve, Heng-shi-tang Station, 2021.V.29, 24.355035°N, 113.399182°E, 332m, leg. Yongying Ruan. • 2 individuals, China, **Guangdong**, Zhaoqing, Mt. Ding-hu-shan, 2021.V.15, leg. Haidong Yang. • 1 individual, China, **Jiangxi**,

Ganzhou, Wu-dang Town, Long-zi-tou, 2021.VI.7, leg. Yongying Ruan. • 2 individuals, China, **Guangdong**, Shao-guan, Nan-ling National Nature Reserve, Babaoshan Station, 2021.V.17, 24.932359°N, 113.019142°E, 1000m, leg. Yongying Ruan, light trap. • 6 individuals, China, **Guangdong**, Qingyuan, Shi-men-tai National Nature Reserve, Qianjin Station, 2021.V.25, 24.488679°N, 113.114489°E, 524m, leg. Yongying Ruan.

Campsosternus gemma Candèze: • 1 specimen, China, **Hunan**, Huaihua, Chen-xi County, 2021.VII.18, leg. Gang Huang.

Actenicerus maculipennis (Schwarz): 1 individual, China, **Guangdong**, Shao-guan, Nan-ling National Nature Reserve, Babaoshan Station, 2021.V.17, 24.932359°N, 113.019142°E, 1000m, leg. Yongying Ruan, light trap. • 1 individual, China, **Guangdong**, Shao-guan, Nan-ling National Nature Reserve, Babaoshan Station, 2021.V.17, 24.932359°N, 113.019142°E, 1000m, leg. Yongying Ruan, light trap.

Ampedus sp.: 1 individual, China, **Hunan**, Chen-zhou, Mang-shan National Nature Reserve, Jiang-jun-zhai, 2021.V.23, 24.951510°N, 112.982358°E, 1300m, leg. Yongying Ruan.

Cardiophorus sp.: 1 individual, China, **Guangdong**, Shao-guan, Nan-ling National Nature Reserve, Babaoshan Station, 2021.V.17, 24.932359°N, 113.019142°E, 1000m, leg. Yongying Ruan, light trap.

Cryptalaus larvatus (Candèze): 1 individual, China, **Guangdong**, Shao-guan, Nan-ling National Nature Reserve, Babaoshan Station, 2021.V.17, 24.932359°N, 113.019142°E, 1000m, leg. Yongying Ruan, light trap. • 4 individuals, China, **Hunan**, Chen-zhou, Mang-shan National Nature Reserve, Jiang-jun-zhai, 2021.V.23, 24.951510°N, 112.982358°E, 1300m, leg. Yongying Ruan.

Ludioschema dorsale (Candèze, 1878): 1 individual, China, **Guangdong**, Shaoguan, Che-ba-ling National Nature Reserve, Chebaling Village, 2021. VI.2, 24.703638°N, 114.151580°E, 487m, leg. Yongying Ruan

Ludioschema obscuripes (Gyllenhal): 1 individual, China, **Guangdong**, Shao-guan, Nan-ling National Nature Reserve, Babaoshan Station, 2021.V.17, 24.932359°N, 113.019142°E, 1000m, leg. Yongying Ruan, light trap. • 1 individual, China, **Guangdong**, Qingyuan, Shi-men-tai National Nature Reserve, Qianjin Station, 2021.V.25, 24.488679°N, 113.114489°E, 524m, leg. Yongying Ruan. • 3 individuals, China, **Guangdong**, Shaoguan, Che-ba-ling National Nature Reserve, Chebaling Village, 2021. VI.2, 24.703638°N, 114.151580°E, 487m, leg. Yongying Ruan. • 6 individuals, China, **Guangdong**, Shenzhen, 2021.V. leg. Yongying Ruan.

Melanotus sp.: 1 individual, China, **Guangdong**, Shao-guan, Nan-ling National Nature Reserve, Babaoshan Station, 2021.V.17, 24.932359°N, 113.019142°E, 1000m, leg. Yongying Ruan, light trap. • 7 individuals, China, **Guangdong**, Shaoguan, Che-ba-ling National Nature Reserve, Chebaling Village, 2021. VI.2, 24.703638°N, 114.151580°E, 487m, leg. Yongying Ruan. • 10 individuals, China, **Hunan**, Chen-zhou, Mang-shan National Nature Reserve, Jiang-jun-zhai, 2021.V.23, 24.951510°N, 112.982358°E, 1300m, leg. Yongying Ruan.

Pectocera fortunei Candèze: 2 individuals, China, **Hunan**, Chen-zhou, Mang-shan National Nature Reserve, Jiang-jun-zhai, 2021.V.23, 24.951510°N, 112.982358°E, 1300m, leg. Yongying Ruan.

Priopus angulatus (Candèze): 1 individual, China, **Guangdong**, Shaoguan, Che-ba-ling National Nature Reserve, Chebaling Village, 2021.VI.2, 24.703638°N, 114.151580°E, 487m, leg. Yongying Ruan

Priopus sp.: 1 individual, China, **Guangdong**, Shao-guan, Nan-ling National Nature Reserve, Babaoshan Station, 2021.V.17, 24.932359°N, 113.019142°E, 1000m, leg. Yongying Ruan, light trap.

Silesis sp.: 3 individuals, China, **Guangdong**, Qingyuan, Shi-men-tai National Nature Reserve, Qianjin Station, 2021.V.25, 24.488679°N, 113.114489°E, 524m, leg. Yongying Ruan.

Sinelater perroti (Fleutiaux): • 1 individual, China, **Guangxi**, Guilin, Zi-yuan County, 2021.VI.21, leg. Wenyu Ma, light trap. • 1 individual, China, **Guangxi**, Guilin, Zi-yuan

County, 2021.VII.7, leg. Wenyu Ma, light trap.

Sternocampsus coriaceus Liu *et* Jiang: 1 individual, China, **Guangdong**, Shao-guan, Nan-ling National Nature Reserve, Babaoshan Station, 2021.V.17, 24.932359°N, 113.019142°E, 1000m, leg. Yongying Ruan, light trap. • 1 individual, China, **Hunan**, Chen-zhou, Mang-shan National Nature Reserve, Jiang-jun-zhai, 2021.V.23, 24.951510°N, 112.982358°E, 1300m, leg. Yongying Ruan.

(5) Specimens used for the recording of the clicking sounds.

Campsosternus auratus (Drury): 2 individuals, China, **Guangxi**, Guilin, Zi-yuan County, 2021.VII.15, leg. Wenyu Ma, light trap. • 1 individual, China, **Fujian**, Nanping, Shao-wu, 2021.VII.15, leg. Chen Chen.

(6) The specimen that was used for an additional test to observe the displacement of the mesonotum in the loading phase.

Campsosternus auratus (Drury): 1 individual, China, **Fujian**, Fuan, Shuyang Village, 368 m, 27°09′32″N,119°40′34″E, 2021. VII.15, leg. Xiuzhao Lin.

Sinelater perroti (Fleutiaux): 1 individual, China, **Guangxi**, Guilin, Zi-yuan County, 2021.VII.15, leg. Wenyu Ma, light trap.

(7) The specimen that was used for observation of the interlocking mechanism of the thorax

Campsosternus auratus (Drury) (Elateridae): 4 individuals, China, **Fujian**, Nanping, 2021.VII.14, leg. Mengna Zhang.

Sinelater perroti (Fleutiaux) (Elateridae): 1 individual, China, **Guangdong**, Shao-guan, Che-ba-ling National Nature Reserve, 2020.VIII.23., Yongying Ruan, light trap.

Callirhipis sp. (Callirhipidae): 2 individuals, China, **Guangdong**, Shao-guan, Nan-ling National Nature Reserve, Babaoshan Station, 2021.V.17, 1000m, leg. Yongying Ruan, light trap.

Eulichas cf. *funebris* (Westwood) (Eulichadidae): 2 individuals, China, **Guangxi**, Guilin, Zi-yuan County, 2021.VII.15, leg. Wenyu Ma, light trap.

Chalcophora yunnana Fairmaire (Buprestidae): 5 individuals, China, **Guangxi**, Guilin, Zi-yuan County, 2021.VII.15, leg. Wenyu Ma.
