# Supplementary material for: Functional Morphology of the Thorax of the Click Beetle Campsosternus auratus (Coleoptera, Elateridae), with an Emphasis on Its Jumping Mechanism
Source: Insects. 2022 Feb 28;13(3):248. doi: 10.3390/insects13030248 (PMC8955093; doi:10.3390/insects13030248)
Supplement: Supplementary file 1 [file insects-13-00248-s001.zip › Supplementary file S2. Simplified 3D models of the exoskeleton and thoracic muscles of Campsosternus auratus.pdf]

Interactive 3D PDF

Instructions on the use of this interactive 3D PDF

1. Click on the object to activate the interaction with the object.
2. Interaction tools in 3D toolbar enable rotation and resizing of the 3D object. 3D toolbar is available at the top of each page.
   Rotate: Hold left mouse button and move mouse.
   Zoom: Scroll mouse wheel.
   Translate: Hold left and right mouse buttons and move mouse.
   Cut: Use the 'cutting tool' to cut the model and visualize the internal musculature and structures.

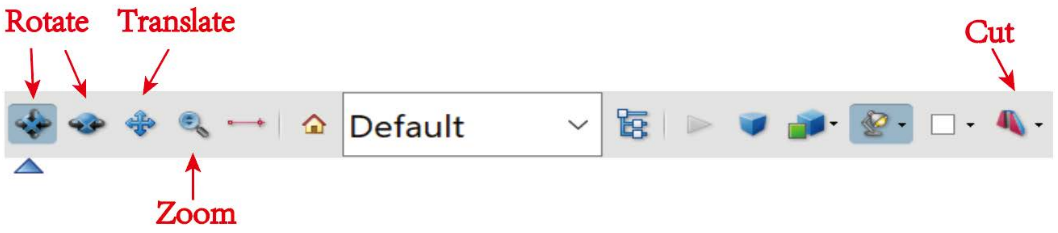

SOLIDWORKS / Composer

Exoskeleton and thoracic muscles of *Campsosternus auratus*

Requirements

This installation requires Adobe Acrobat Reader 7.0 or Adobe Acrobat 7.0

You must install one of these products before installation of the plug-in. Adobe products are able on Adobe web site:

www.adobe.com

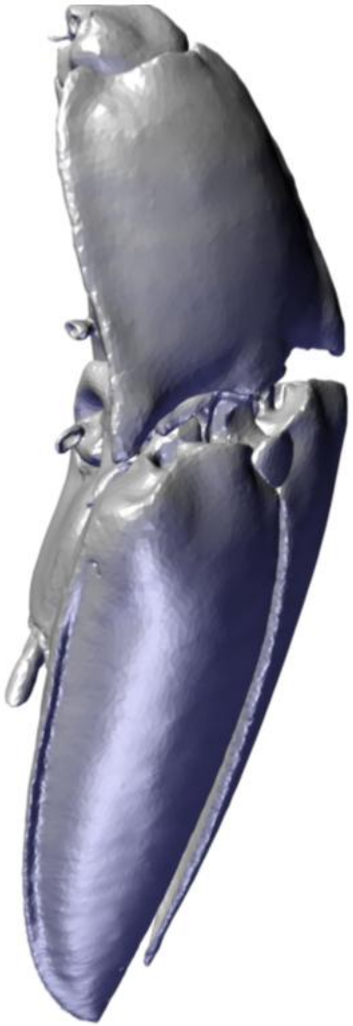

info@solidworks.com

SOLIDWORKS / Composer

Thoracic exoskeleton of *Campsosternus auratus*

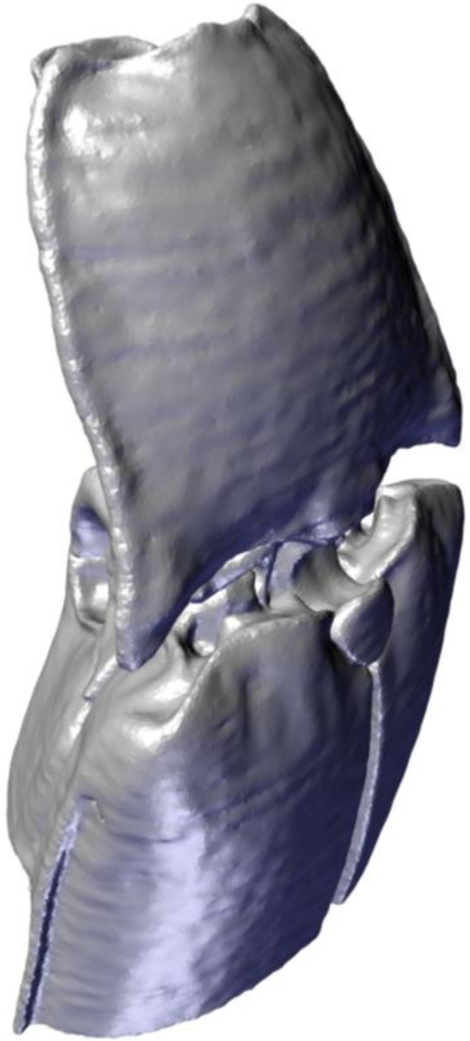

Requirements

This installation requires Adobe Acrobat Reader 7.0 or Adobe Acrobat 7.0

You must install one of these products before installation of the plug-in. Adobe products are able on Adobe web site:
www.adobe.com

info@solidworks.com

SOLIDWORKS / Composer

Meso- and metathoracic exoskeleton of *Campsosternus auratus*

Requirements

This installation requires Adobe Acrobat Reader 7.0 or Adobe Acrobat 7.0

You must install one of these products before installation of the plug-in. Adobe products are able on Adobe web site:
www.adobe.com

info@solidworks.com

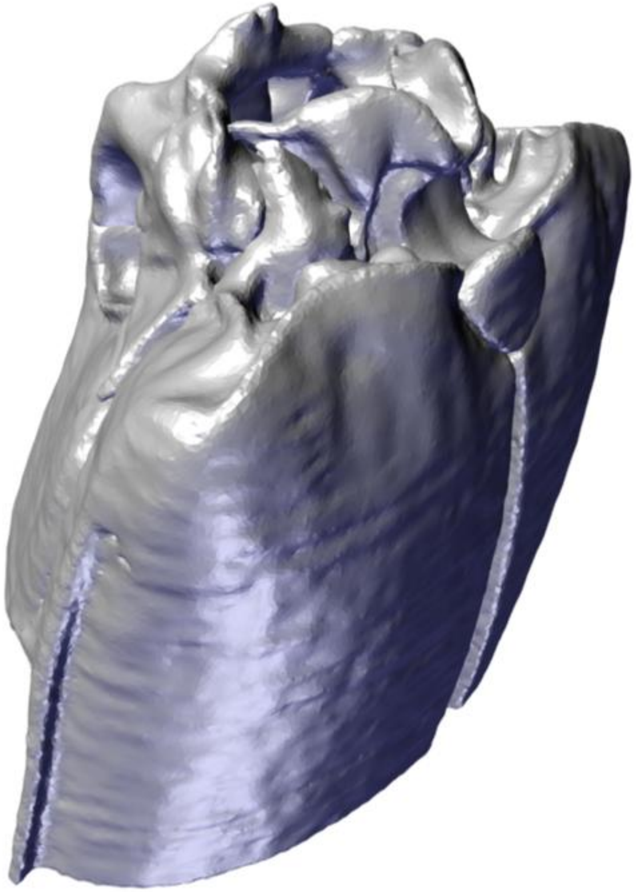

SOLIDWORKS / Composer

Meso- and metathoracic exoskeleton (elytra removed) of *Campsosternus auratus*

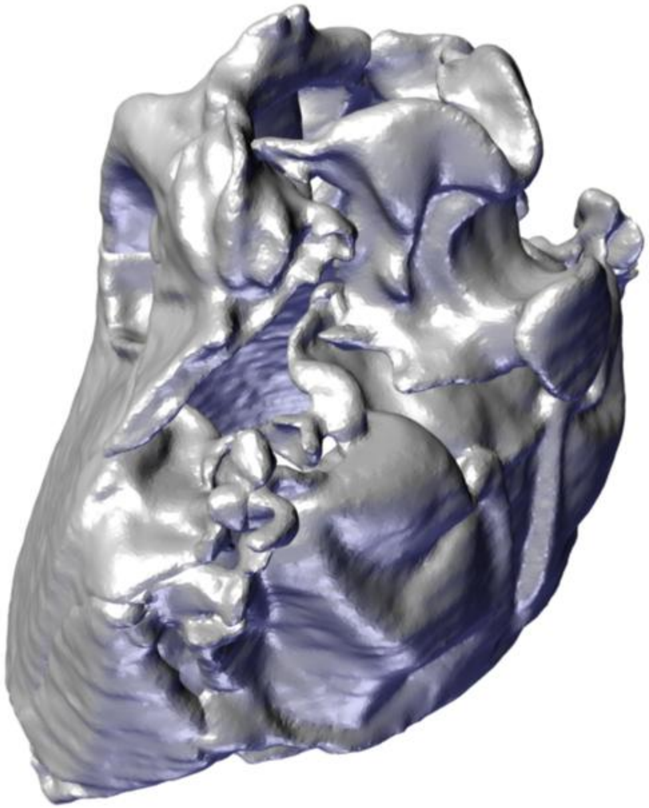

Requirements

This installation requires Adobe Acrobat Reader 7.0 or Adobe Acrobat 7.0

You must install one of these products before installation of the plug-in. Adobe products are able on Adobe web site:

- **www.adobe.com**

SOLIDWORKS / Composer

Mesonotum of *Campsosternus auratus*

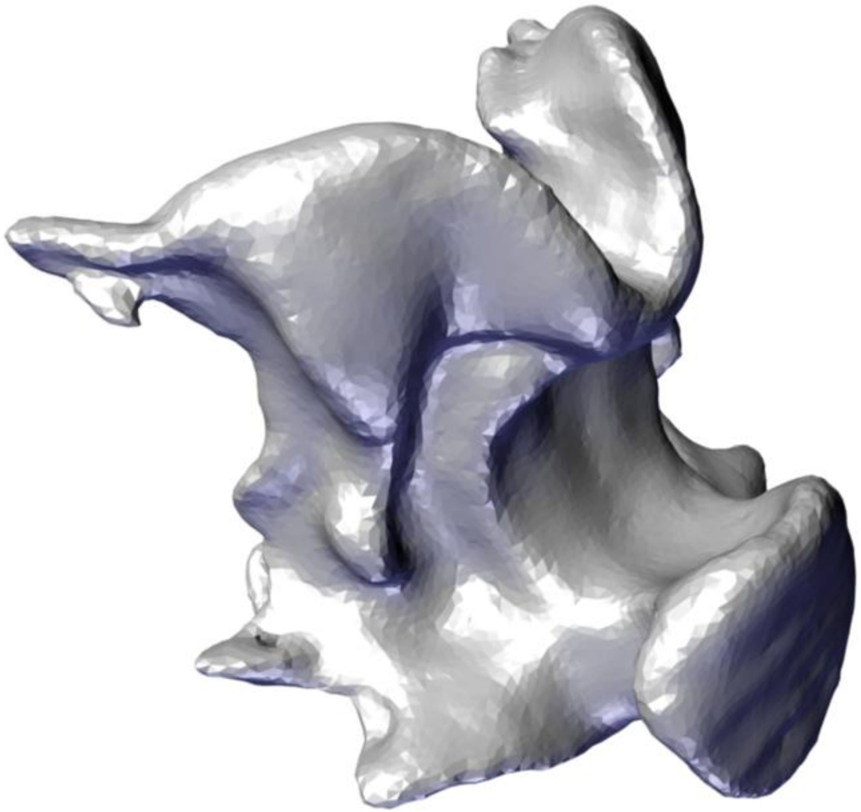

Requirements

This installation requires Adobe Acrobat Reader 7.0 or Adobe Acrobat 7.0

You must install one of these products before installation of the plug-in. Adobe products are able on Adobe web site:
www.adobe.com

info@solidworks.com

SOLIDWORKS / Composer

Exoskeleton of prothorax of *Campsosternus auratus*

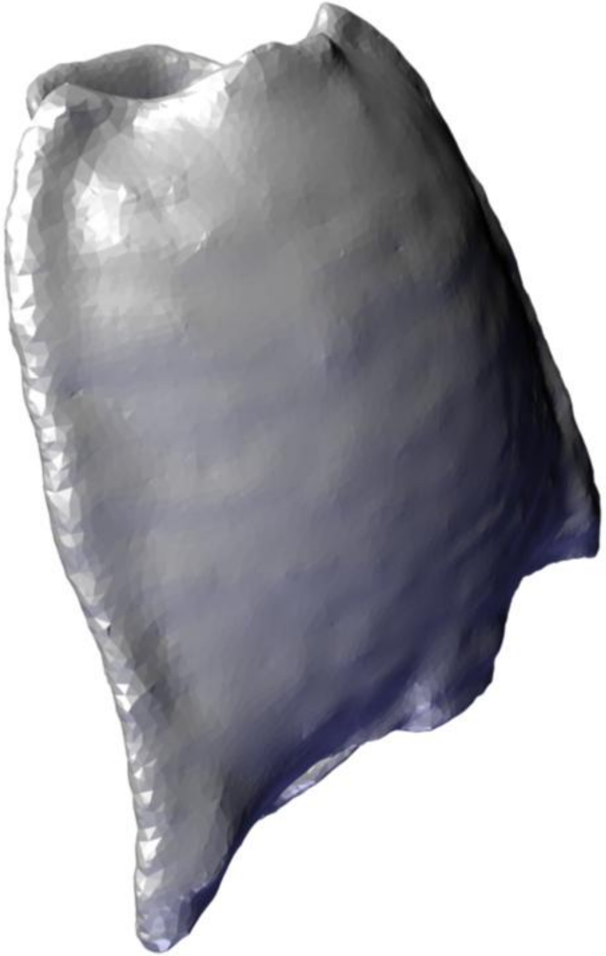

Requirements

This installation requires Adobe Acrobat Reader 7.0 or Adobe Acrobat 7.0

You must install one of these products before installation of the plug-in. Adobe products are able on Adobe web site:

www.adobe.com

info@solidworks.com
